# Offshore wind power station (OWPS) site selection using a two-stage MCDM-based spherical fuzzy set approach

**DOI:** 10.1038/s41598-022-08257-2

**Published:** 2022-03-11

**Authors:** Chia-Nan Wang, Ngoc-Ai-Thy Nguyen, Thanh-Tuan Dang

**Affiliations:** 1 Department of Industrial Engineering and Management, National Kaohsiung University of Science and Technology, Kaohsiung, 80778 Taiwan; 2 Department of Logistics and Supply Chain Management, Hong Bang International University, Ho Chi Minh, 723000 Vietnam

**Keywords:** Climate sciences, Energy science and technology, Engineering, Mathematics and computing

## Abstract

In response to challenges from the COVID-19 pandemic and climate change to achieve the goal of ensuring sustainable economic growth, offshore wind power development not only provides a clean and sustainable source of energy but also provides opportunities for economic growth and job creation. Offshore wind energy projects have been promptly suggested in Vietnam due to policy advancement, with the country's excellent wind resources. The success of an offshore wind energy project is decided mainly by choosing the best location for offshore wind power station (OWPS) construction, which is a complex multicriteria decision-making (MCDM) problem with the coexistence of conflicting factors. There is a problem with incomplete decision information use and information loss during the decision-making process, and it is easy to overlook the interaction difficulty in a fuzzy environment. To address the complex nature of the prioritization problem posed, this study proposes a hybrid MCDM framework combining the spherical fuzzy analytical hierarchy process (SF-AHP) and weighted aggregated sum product assessment (WASPAS). SF-AHP is used in the first stage to determine the significance levels of OWPS evaluation criteria. WASPAS is then utilized to rank locations of OWPS. A comprehensive set of evaluation criteria developed based on the concept of sustainable development has been recognized by reviewing the literature review and interviewing experts to practice the two-stage MCDM model. A real case study for Vietnam is conducted to test the effectiveness of the proposed method. The best location schemes have been determined by using the decision framework. The results of the sensitivity analysis and a comparison analysis demonstrate that the decision framework is practical and robust. The proposed methodology can be used to attain a decision-making process at the regional level for offshore wind farm planning and coastal development, and the study results encourage the establishment of renewable energy development policies.

## Introduction

Among various renewable energy sources, offshore wind is key to the transition to a zero-carbon energy supply in the context of the whole world facing the global fight against climate change and promoting a post-COVID-19 green recovery. According to the International Renewable Energy Agency (IRENA), the world needs to install at least 180 GW of new wind power each year to keep the global temperature rise below 2 °C above preindustrial levels^1^. Located in the monsoon climate zone and shaped by a 3260 km-long coastline, Vietnam is considered a country with great potential for offshore wind power in Asia in particular and the world in general. As reported by the World Bank, Vietnam’s technical offshore wind power potential is approximately 475 GW in water zones 200 km from the coastline, and the technical offshore wind power potential in the water zones ranges from 0 to 185 km up to 600 GW^2^. With this endowment, Vietnam can achieve 11 GW to 25 GW of offshore wind capacity by 2035, which could create up to 700,000 jobs per year and reduce 217 million tons of carbon emissions.

Under the Vietnamese government's new Power Development Plan with a vision to 2030, a 20 GW of renewable energy capacity, including 10 GW of offshore wind power, aims to meet growing demand and sustainable socioeconomic development and cut 15% of carbon emissions^3^. Toward this goal, experts and researchers assert that policies and support mechanisms play an essential role in building national strategies and marine spatial planning for offshore wind power development. A study by Xuan Son and Thi Gam in 2021^4^ analyzed the current Vietnamese legal and policy framework applying offshore wind power development and Vietnamese regulations to assess the environmental impact to guarantee sustainable wind development. More specifically, prioritizing capacity development in line with the ability to ensure power system safety through reasonable electricity tariffs enables breakthrough supportive policies and mechanisms for offshore wind power development. Nguyen et al.^5^ focused on various challenges of offshore wind energy development in Vietnam, such as policy and regulatory uncertainty, construction costs, installations, sea area planning, and high investment risks. To exploit the potential of offshore wind, it is vital to pay attention to some specific characteristics because of wind's dependence on climatic and geographical conditions, and wind regimes are unevenly spread across the country. If harnessed effectively and sustainably, offshore wind can provide numerous benefits, including the valuable resource of potentially lower energy costs, the security of long-term supply of both electricity and gas, the creation of jobs and supply chain opportunities, and the improvement of environmental quality^6^. There have been few studies of Vietnam's offshore wind potential^7–9^. Studies on the location decision process for offshore wind projects are also limited.

Site selection for an offshore wind power station (OWPS) constitutes a critical phase toward a wind power project. It is a multicriteria decision-making (MCDM) problem regarding many conflicting criteria, including wind resources, construction, environmental impacts, marine spatial planning, power grid access lines, economy, and society^10^. With this, the decision-making of OWPS site selection encounters many difficulties. One primary concern is the ambiguity of information. It is a daunting problem to anticipate the value of each factor precisely during decision-making as a project prework. To a large degree, the stated judgments of specialists (experts) tend to be vague due to linguistic terms that an exact value cannot express; thus, information loss during the evaluation process is an unavoidable occurrence in a complex and fuzzy context. As a result, how information is expressed and handled is an essential consideration in OWPS site selection. As soon as ranking methods are becoming increasingly improved, using an adequate and effective MCDM method to determine the priority of alternatives in OWPS site selection is a requisite step. Apart from the numerous extensions of fuzzy linguistic term sets, several methods have been designed due to the diverse nature of MCDM problems. Some distinctive approaches among them are VIšeKrite-rijumska Optimizacija I Kompromisno Rešenje (VIKOR), a technique for order of preferences by similarity to ideal solution (TOPSIS), complex proportional assessment (COPRAS), weighted aggregated sum product assessment (WASPAS), to name a few. For instance, Shemshadi et al.^11^ extended the VIKOR method to extract and deploy objective weights based on the Shannon entropy concept to handle supplier selection problems. According to Mousavi-Nasab and Sotoudeh-Anvari^12^, TOPSIS and COPRAS have been effective methods in the material selection problem in general practice. Garg and Kumar^13^ developed the TOPSIS method based on novel exponential distance measures with set pair analysis theory for the interval-valued intuitionistic fuzzy set to cope with real-world problems. Among these methods, WASPAS is a relatively novel and straightforward MCDM method that has been broadly applied in several practical problems^14–17^.

This paper aims to develop an MCDM-based framework for the best site selection of OWPSs. More specifically, the spherical fuzzy sets and analytical hierarchy process (SF-AHP) are integrated into the first stage to determine the significance levels of OWPS evaluation criteria, and then the weighted aggregated sum product assessment (WASPAS) is utilized to rank locations of OWPS. A comprehensive set of evaluation criteria developed based on the concept of sustainable development has been recognized through a literature review and expert opinions to practice the two-stage MCDM model. A real case study for Vietnam is carried out to validate the proposed method.

**Figure 1:**
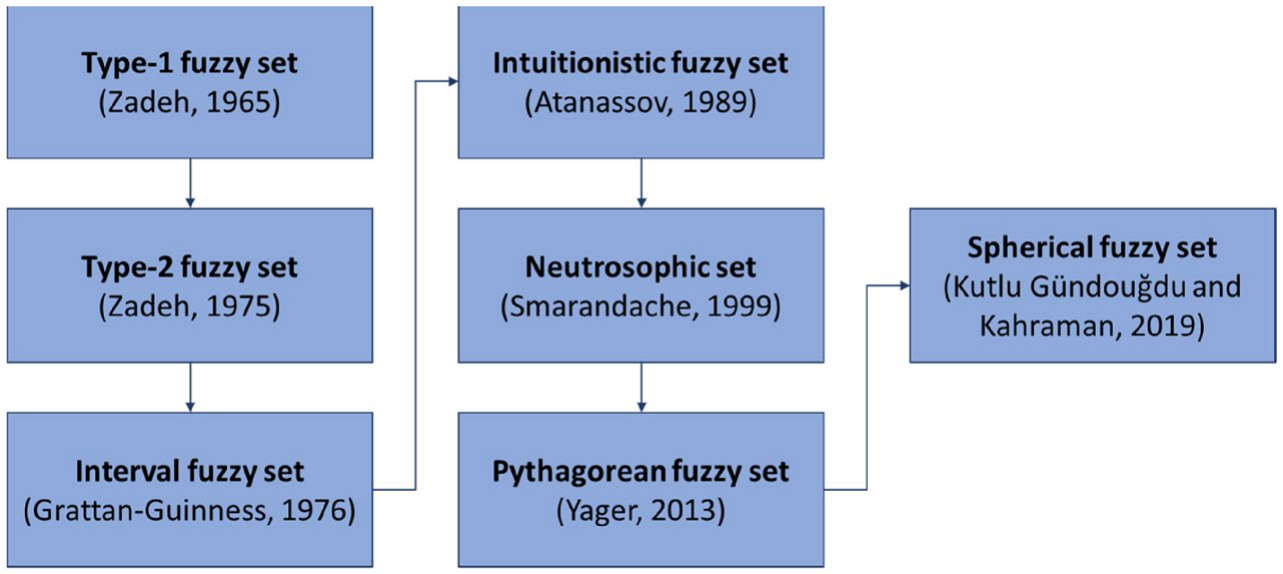
The historical mapping of extensions of fuzzy sets.

The AHP is a relative measurement method that can rank multiple alternatives by examining both qualitative and quantitative criteria based on pairwise comparisons. The method is one of the most commonly used MCDM methods to determine the relative importance (weights) of criteria and subcriteria, especially in renewable energy planning and site selection^10^. Even while the approach gathers data from experts, it may not precisely reflect the opinions taken. As a result, fuzzy sets theory has been integrated with AHP, and many types of fuzzy AHP have been developed to capture vagueness in preference. The effectiveness of fuzzy AHP methods has been demonstrated with increasing interest among researchers and practitioners. Such approaches have been implemented on different extensions of fuzzy set theory based on the determination of linguistic statements such as traditional fuzzy sets^18–20^, type-2 fuzzy sets^21,22^, interval-valued fuzzy sets^23–25^, intuitionistic fuzzy sets^26–28^, neutrosophic sets^29,30^, Pythagorean fuzzy sets (PSF)^31,32^, and spherical fuzzy sets^33,34^. The spherical fuzzy set (SFS) is the novel set introduced in 2018 by Kutlu Gündoğdu and Kahraman^35–38^. It is a three-dimensional fuzzy set created as a combination of Pythagorean fuzzy sets with neutrosophic fuzzy sets. SFS can also be used to realize the criteria to handle ambiguity and fuzziness in linguistic expressions, which is a new perspective for decision-making in a fuzzy environment. The decision maker's indeterminacy level is specified independently of the membership and nonmembership levels of the elements in these sets. Decision-makers define the membership function in SFS on a spherical surface to infer other fuzzy sets, with which they can allow the parameters of this membership function in a broader domain. The historical mapping of different fuzzy set extensions is displayed in Fig. 1^35,39–44^.

WASPAS was first proposed in 2012 by Zavadskas, Turskis, Antucheviciene, and Zakarevicius^45^ and is one of the robust new MCDM utility determining approaches. The method integrates the weighted product model (WPM) and weighted sum model (WSM), which the authors have proven to have more robust consistency and accuracy than the WPM and WSM. It also performs more accurately than independent methods in alternative ranking^45^. Ever since this aggregated approach appeared, a plethora of studies can be found using WASPAS in various areas. In 2013, Zolfani et al.^46^ applied WASPAS to business issues with a case study of selecting the best place for shopping malls located from a foresight perspective; Zavadskas et al.^47^ utilized the method to evaluate facade alternatives; Bagočius et al.^48^ used WASPAS for the selection of a deep-water port. WASPAS was employed by Vafaeipour et al.^49^ for the region priority problem for solar project implementation. Badalpur and Nurbakhsh^50^ evaluated the risks of a road construction project by taking the merits of the WASPAS method. To solve the supplier selection problem, Singh and Modgil^51^ used WASPAS to finalize the best supplier in the cement industry. Considering the increasing trend of the delivery industry, in the most recent studies, Nguyen et al.^52^ utilized WASPAS to select the best online food delivery companies; Wang et al.^53^ used the method to evaluate sustainable last-mile delivery for e-commerce companies.

The paper’s contributions are presented as follows:

- This paper presents an effective evaluation model for locating offshore wind power facilities. To fulfill the awareness of sustainable development, the model contains a comprehensive set of sustainability indicators.
- The calculations for weighting the criteria are performed using spherical fuzzy sets for a broader linguistic scale of experts’ judgments, which completely reflects the decision-making process in uncertain environments. WASPAS has the capability and more accuracy in ranking the alternatives.
- A thorough investigation of the OWPS site selection in Vietnam is solved for the first time, with a real case study used to test the robustness of the proposed model.
- The paper is directed toward providing a recommendation for the government and practitioners for offshore wind farm site selection.

The remainder of this paper is structured as follows. “Literature review” summarizes MCDM methodologies applied to the site selection of OWPS and critical evaluation criteria used in the studies. In “Materials and methods”, the implementation of the proposed hybrid methodology is explained in detail. In “Results analysis”, the OWPS case study analysis in Vietnam is demonstrated, and then the results validation is conducted in “Results validation”. “Managerial implications” contains concluding remarks.

## Literature review

### Literature review on OWPS selection and evaluation

There have been a moderate number of significant studies on OWPS selection in the last ten years, in which MCDM approaches have shown remarkable results in many case evaluations worldwide. Some widely applied techniques in OWPS evaluation include analytic hierarchy process (AHP), analytic network process (ANP), technique for order of preference by similarity to ideal solution (TOPSIS), elimination and choice expressing the reality (ELECTRE), decision making trial and evaluation laboratory (DEMATEL), preference ranking organization method for enrichment evaluations (PROMETHEE). Fuzzy sets theory and grey theory are frequently integrated with MCDM methods to address uncertain and incomplete information/preference. Chaouachi et al.^54^ presented the multicriteria selection of offshore wind farms with a case study for the Baltic States; the AHP method was utilized to consider economic investment, security aspects, operation costs and capacity performances. Fetanat and Khorasaninejad^19^ developed a novel hybrid MCDM approach based on the fuzzy ANP, fuzzy DEMATEL, and fuzzy ELECTRE to assist in the site selection of offshore wind farms in Iran; six criteria (depth and height, environment, distance to facilities, economic aspects, wind resources, and culture) were determined with related subcriteria. A decision framework combining triangular intuitionistic fuzzy numbers (TIFNs), ANP and PROMETHEE was proposed by Wu et al.^28^ to select the best location for OWPS in a Chinese case study considering six criteria (wind resources, environment, economic, construction, society, and risks) and the related subcriteria. Lo et al.^55^ proposed the grey DEMATEL-based ANP model for location optimization for OWPSs in Taiwan, concerning the following dimensions: wind conditions, marine conditions, shore support conditions, economic impacts, environmental and ecological impacts, and societal impacts. Table 1 provides an overview of studies on the site selection of offshore wind farms.

**Table 1:** Overview of studies on the site selection of offshore wind farms.

No.	Authors	MCDM Technique	Location	Main findings
1	Fetanat and Khorasaninejad^19^	Fuzzy ANP, fuzzy DEMATEL, and fuzzy ELECTRE	Iran	The optimal site can be chosen from four options, and the method's robustness is proven
2	Wu et al.^27^	ELECTRE-III	China	The developed methodology for OWPS site selection is both valid and practical
3	Vasileiou et al.^56^	AHP	Greece	The finding illustrates the potential for offshore wind and wave energy deployment in Greece, particularly in Crete's offshore areas and a longitudinal zone extending from the north-central to the central Aegean
4	Chaouachi et al.^54^	AHP	Baltic States	The best wind sites are determined by market design, regulatory considerations, and renewable integration targets
5	Mahdy and Bahaj^57^	AHP	Egypt	The established methodology is universal to produce offshore wind suitability map for appropriate offshore wind locations, with three high wind suitable areas around the Red Sea found with the minimum restrictions
6	Wu et al.^58^	Fuzzy AHP	China	The approach is applied to a real-world site selection of offshore wind farms in the Eastern China Sea; it illustrates that maritime safety is a predominant factor
7	Emeksiz and Demirci^59^	AHP	Turkey	Analysis of wind resources and regulations is key to offshore wind farm planning and development at the regional level
8	Wu et al.^28^	Fuzzy ANP-PROMETHEE	China	The decision model proposed is feasible and valid
9	Abdel-Basset et al.^60^	AHP and PROMETHEE-II	Egypt	Rigorous methodological support is presented for site selection to achieve benefits in coastal management
10	Lo et al.^55^	Grey DEMATEL-based ANP	Taiwan	Optimal sites are not only determined by their wind resources and costs; decision-makers must pay particular attention to appropriate strategies and policy planning toward OWPS

Since investment is huge in the installment of OWPSs, it is critical to identify and prioritize viable locations prior to developing expensive OWPSs, as such decisions would assist in achieving the best productivity, reducing socioeconomic costs, minimizing environmental consequences, optimizing social benefits, and developing the concerned regions toward sustainability. The recognition of resources, conditions, economic and environmental dimensions, societal impacts, and political factors is the preliminary and crucial phase for establishing new offshore wind plants. Only a few have taken sustainability issues into account from previous works when devising a site selection framework for offshore wind farms. In this research, the decision criteria based on the perspective of sustainability are extracted through a literature review and recognized by experts, as shown in Table 2.

**Table 2:** Summary of criteria considered from the literature reviewed.

Criteria	Fetanat and Khorasaninejad^19^	Wu et al.^27^	Vasileiou et al.^56^	Chaouachi et al.^54^	Mahdy and Bahaj^57^	Wu et al.^58^	Wu et al.^28^	Abdel-Basset et al.^60^	Lo et al.^55^
Wind resources	v	v	v	v	v	v	v	v	v
Seawater depth	v	v	v	v	v	v		v	v
Undersea geological conditions		v					v	v	v
Marine conditions	v	v				v	v	v	v
Environmental protection	v	v		v			v	v	v
Distance to shore	v	v	v	v	v	v		v	v
Electricity networks	v	v	v	v				v	
Traffic condition		v	v		v	v	v	v	v
Profit	v	v					v	v	v
Construction, operation, and maintenance costs		v					v	v	v
Local subsidies		v							
Job creation		v					v	v	v
Policy planning							v	v	v

### Research gaps

From the review, it is deduced that ample literature on OWPS location selection is missing, especially in the Vietnamese context. Keeping in view the same, efforts have been made for the first time in the present study that takes the merits of spherical fuzzy sets, AHP and WASPAS to identify the most suitable sites for OWPS construction. While the AHP method is classical and one of the most effective techniques for MCDM problems, both spherical fuzzy and WASPAS are among the most novel methodologies which have multiple advantages, as presented earlier; thus, one of the motivations of this research is to display the applicability of these two methods than other fuzzy MCDM approaches for the OWPS site selection. The suggested MCDM approach has the advantage of processing the uncertain evaluations expressed in the form of spherical fuzzy environments that do not omit any information from human judgments and generate a more accurate and robust ranking for the alternatives with the novel WASPAS method. In fact, to the best knowledge of the authors, the present investigation is the first attempt to use SF-AHP/WASPAS analysis for OWPS site selection in Vietnam and is missing in the existing literature of renewable energy site selection.

Thus, to fulfill the research gap, initially, the important key factors influencing OWPS site selection were identified from the literature and validated in discussion with experts. Later, SF-AHP and WASPAS methodologies were utilized to select the best sites based on the evaluation indicator system, including wind resources, environmental impact, construction and maintenance conditions, societal impact, conditions onshore, and economic impact.

## Materials and methods

### Framework of the research

**Figure 2:**
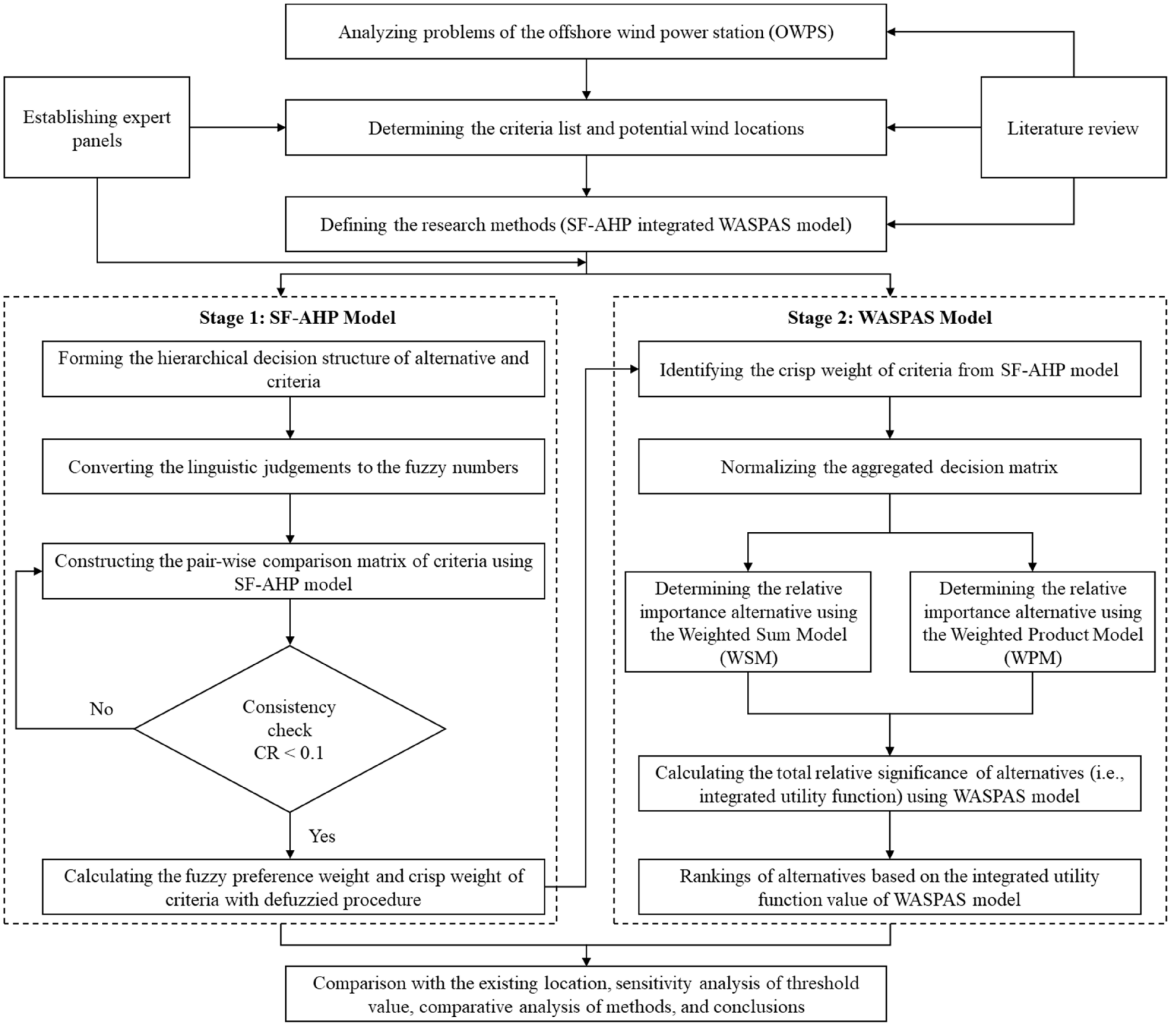
The proposed framework of the research.

This paper introduces an effective integrated assessment model for evaluating and selecting the optimal OWPS case study in Vietnam. The proposed framework includes two stages, which are described in Fig. 2. In the first stage, the SF-AHP model determines each criterion's fuzzy weight and crisp weight. The spherical fuzzy set, represented by linguistics number, is involved in the AHP model to manage the uncertainties and vagueness by the experts' judgment. The consistency of the pairwise comparison matrices is checked to ensure the model's validation. In the second stage, the wind power locations are ranked by using the WASPAS model. Next, a comparison with the existing area, sensitivity analysis of the threshold value, and comparative analysis of the methods are performed to demonstrate the feasibility and applicability of the proposed model.

### Spherical fuzzy analytical hierarchy process (SF-AHP)

**Figure 3:**
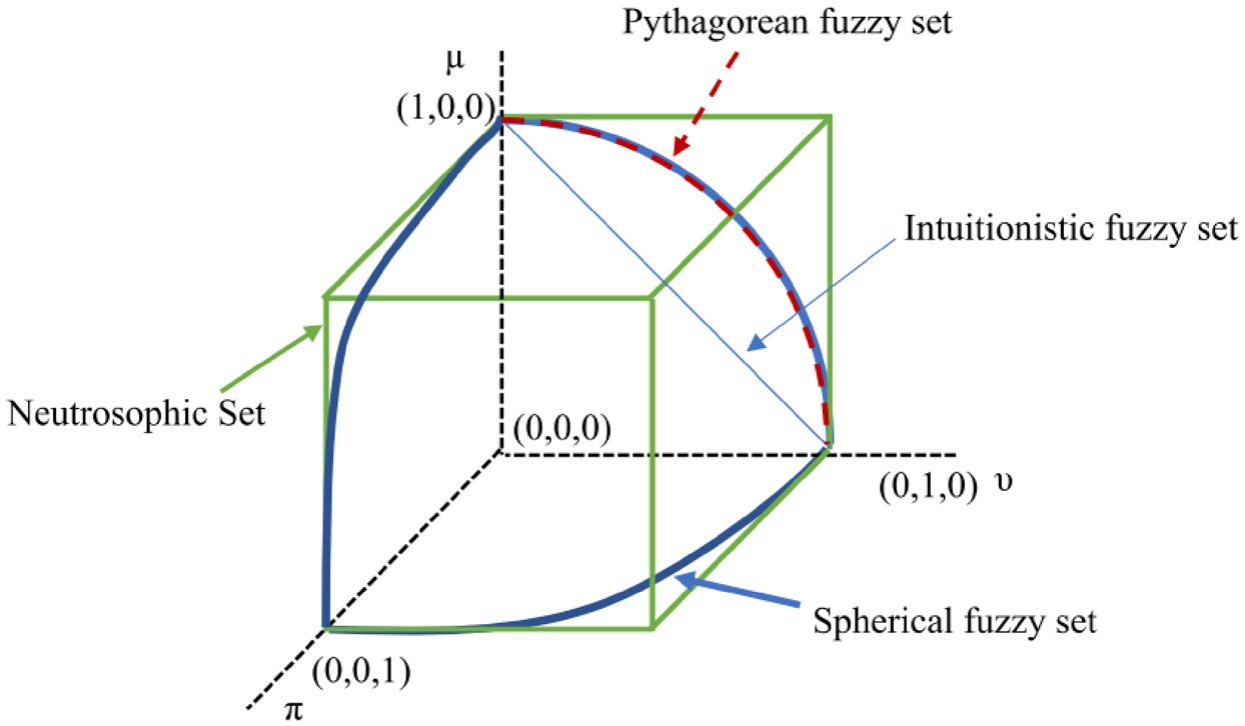
Geometric representations of spherical fuzzy sets in 3D space.

Spherical fuzzy sets (SFS) were newly developed by Kutlu Gündouğdu and Kahraman^38^ to handle uncertainty during the quantification of expert judgments. The differences among the intuitionistic fuzzy set, Pythagorean fuzzy set, neutrosophic set, and spherical fuzzy sets^33^ are visualized in Fig. 3. The SFS consists of three parameters: membership, nonmembership, and hesitancy degrees. The basic procedures of SFS are presented as follows.

#### Definition 1

Spherical fuzzy set (SFS) ${\tilde{A }}_{S}$ is described as follows:

1 $${\tilde{A }}_{S}=\left\{x,\left({\mu }_{{\tilde{A }}_{S}}\left(x\right), {v}_{{\tilde{A }}_{S}}\left(x\right), {\pi }_{{\tilde{A }}_{S}}\left(x\right)\right)|x\in X\right\},$$

where ${\tilde{A }}_{S}$ represents a spherical fuzzy set of the universe $X$:

2 $${\mu }_{{\tilde{A }}_{S}}\left(x\right): X\to \left[\mathrm{0,1}\right],{v}_{{\tilde{A }}_{S}}\left(x\right):X\to \left[\mathrm{0,1}\right],{\pi }_{{\tilde{A }}_{S}}(x):X\to \left[\mathrm{0,1}\right]$$

and

3 $$0\le {\mu }_{{\tilde{A }}_{S}}^{2}\left(x\right)+{v}_{{\tilde{A }}_{S}}^{2}\left(x\right)+{\pi }_{{\tilde{A }}_{S}}^{2}(x)\le 1,$$

where $\forall x\in X$, and for each $x$, ${\mu }_{{\tilde{A }}_{S}}(x), {v}_{{\tilde{A }}_{S}}(x)$, and ${\pi }_{{\tilde{A }}_{S}}(x)$ represent the membership, nonmembership, and hesitancy levels of $x$ to ${\tilde{A }}_{S}$, respectively.

#### Definition 2

Let ${\tilde{A }}_{S}=({\mu }_{{\tilde{A }}_{S}}, {v}_{{\tilde{A }}_{S}}, {\pi }_{{\tilde{A }}_{S}})$ and ${\tilde{B }}_{S}=({\mu }_{{\tilde{B }}_{S}}, {v}_{{\tilde{B }}_{S}}, {\pi }_{{\tilde{B }}_{S}})$ be two SFS. Some arithmetic operations of SFS are described as follows:

- Union:
  4 $$\begin{aligned} \tilde{A}_{S} \cup \tilde{B}_{S} & = \left\{ {max \left\{ {\mu_{{\tilde{A}_{S} }} , \mu_{{\tilde{B}_{S} }} } \right\},min \left\{ {v_{{\tilde{A}_{S} }} , v_{{\tilde{B}_{S} }} } \right\},}\right. \\&\quad \left.{min \left\{ {\left( {1 - \left( {\left( {max \left\{ {\mu_{{\tilde{A}_{S} }} , \mu_{{\tilde{B}_{S} }} } \right\}} \right)^{2} + \left( {min \left\{ {v_{{\tilde{A}_{S} }} , v_{{\tilde{B}_{S} }} } \right\}} \right)^{2} } \right)} \right)^{1/2} , max \left\{ { \pi_{{\tilde{A}_{S} }} , \pi_{{\tilde{B}_{S} }} } \right\}} \right\}} \right\} \end{aligned}$$
- Intersection:
  5 $${\tilde{A }}_{S} \,\cap \,{\tilde{B }}_{S}= {\{\mathrm{min}\{\mu }_{{\tilde{A }}_{S}}, {\mu }_{{\tilde{B }}_{S}}\},max {\{v}_{{\tilde{A }}_{S}}, {v}_{{\tilde{B }}_{S}}\},\mathrm{ max }\{(1- {{((min \{{\mu }_{{\tilde{A }}_{S}}, {\mu }_{{\tilde{B }}_{S}}\})}^{2}+ {(max {\{v}_{{\tilde{A }}_{S}}, {v}_{{\tilde{B }}_{S}}\})}^{2}))}^{1/2}, min \{ {\pi }_{{\tilde{A }}_{S}}, {\pi }_{{\tilde{B }}_{S}}\}\}\}$$
- Addition:
  6 $${\tilde{A }}_{S} \oplus {\tilde{B }}_{S} = \{{({\mu }_{{\tilde{A }}_{S}}^{2}+{\mu }_{{\tilde{B }}_{S}}^{2} - {\mu }_{{\tilde{A }}_{S}}^{2}{\mu }_{{\tilde{B }}_{S}}^{2})}^{1/2}, {v}_{{\tilde{A }}_{S}}{v}_{{\tilde{B }}_{S}},{((1-{\mu }_{{\tilde{B }}_{S}}^{2}){\pi }_{{\tilde{A }}_{S}}^{2} + (1-{\mu }_{{\tilde{A }}_{S}}^{2}){\pi }_{{\tilde{B }}_{S}}^{2} - {\pi }_{{\tilde{A }}_{S}}^{2}{\pi }_{{\tilde{B }}_{S}}^{2})}^{1/2}\}$$
- Multiplication:
  7 $${\tilde{A }}_{S} \otimes {\tilde{B }}_{S} = \{{\mu }_{{\tilde{A }}_{S}}^{2}{\mu }_{{\tilde{B }}_{S}}^{2},{({v}_{{\tilde{A }}_{S}}^{2}+{v}_{{\tilde{B }}_{S}}^{2} - {v}_{{\tilde{A }}_{S}}^{2}{v}_{{\tilde{B }}_{S}}^{2})}^{1/2},{((1-{v}_{{\tilde{B }}_{S}}^{2}){\pi }_{{\tilde{A }}_{S}}^{2} + (1-{v}_{{\tilde{A }}_{S}}^{2}) {\pi }_{{\tilde{B }}_{S}}^{2} - {\pi }_{{\tilde{A }}_{S}}^{2}{\pi }_{{\tilde{B }}_{S}}^{2})}^{1/2}\}$$
- Multiplication by a scalar; $\lambda >0$*:*
  8 $$\lambda . {\tilde{A }}_{S} = \{{{(1- (1-{\mu }_{{\tilde{A }}_{S}}^{2})}^{\lambda })}^{1/2}, {v}_{{\tilde{A }}_{S}}^{\lambda }, ({(1-{\mu }_{{\tilde{A }}_{S}}^{2})}^{\lambda }- {{(1-{\mu }_{{\tilde{A }}_{S}}^{2}- {\pi }_{{\tilde{A }}_{S}}^{2})}^{\lambda })}^{1/2}\}$$
- Power of $${\tilde{A }}_{S}; \lambda >0$$:
  9 $${\tilde{A }}_{S }^{\lambda }= \left\{{\mu }_{{\tilde{A }}_{S}}^{\lambda }, {\left(1-{\left(1-{v}_{{\tilde{A }}_{S}}^{2}\right)}^{\lambda }\right)}^\frac{1}{2}, {(({1-{v}_{{\tilde{A }}_{S}}^{2})}^{\lambda }-{\left(1-{v}_{{\tilde{A }}_{S}}^{2}-{\pi }_{{\tilde{A }}_{S}}^{2}\right)}^{\lambda })}^\frac{1}{2}\right\}.$$

#### Definition 3

For SFSs ${\tilde{A }}_{S}=({\mu }_{{\tilde{A }}_{S}}, {v}_{{\tilde{A }}_{S}}, {\pi }_{{\tilde{A }}_{S}})$ and ${\tilde{B }}_{S}=({\mu }_{{\tilde{B }}_{S}}, {v}_{{\tilde{B }}_{S}}, {\pi }_{{\tilde{B }}_{S}})$, the following are valid under the condition $\lambda , {\lambda }_{1},{\lambda }_{2}>0$:

10 $${\tilde{A }}_{S} \oplus {\tilde{B }}_{S}={\tilde{B }}_{S} \oplus {\tilde{A }}_{S},$$

11 $${\tilde{A }}_{S} \otimes {\tilde{B }}_{S}={\tilde{B }}_{S} \otimes {\tilde{A }}_{S},$$

12 $$\lambda \left({\tilde{A }}_{S} \oplus {\tilde{B }}_{S}\right)=\lambda {\tilde{A }}_{S} \oplus \lambda {\tilde{B }}_{S},$$

13 $${\lambda }_{1}{\tilde{A }}_{S} \oplus {\lambda }_{2}{\tilde{A }}_{S} = \left({\lambda }_{1}+{\lambda }_{2}\right){\tilde{A }}_{S},$$

14 $$({{\tilde{A }}_{S} \otimes {\tilde{B }}_{S})}^{\lambda }={\tilde{A }}_{S }^{\lambda }\otimes {\tilde{B }}_{S }^{\lambda },$$

15 $${\tilde{A }}_{S }^{{\lambda }_{1}} \otimes {\tilde{A }}_{S }^{{\lambda }_{2}} = {\tilde{A }}_{S }^{{\lambda }_{1}+{\lambda }_{2}}.$$

#### Definition 4

For the spherical weighted arithmetic mean (SWAM) with respect to, $w=({w}_{1}, {w}_{2},..., {w}_{n}{w}_{i}\in [\mathrm{0,1}]$, and ${\sum }_{i=1}^{n}{w}_{i}= 1$*,* the SWAM is calculated as follows:

16 $$\begin{aligned}&{SWAM}_{w}\left({\tilde{A }}_{S1},\dots , {\tilde{A }}_{Sn}\right)={w}_{1}{\tilde{A }}_{S1}+{w}_{2}{\tilde{A }}_{S2}+...+{w}_{n}{\tilde{A }}_{Sn}\\ &\quad=\left\{{\left[1-{\prod\limits_{i=1}^{n}(1- {\mu }_{{\tilde{A }}_{Si}}^{2})}^{{w}_{i}}\right]}^\frac{1}{2}, \prod\limits_{i=1}^{n}{v}_{{\tilde{A }}_{Si}}^{{w}_{i}},{\left[{\prod\limits_{i=1}^{n}(1- {\mu }_{{\tilde{A }}_{Si}}^{2})}^{{w}_{i}}- {\prod\limits_{i=1}^{n}(1- {\mu }_{{\tilde{A }}_{Si}}^{2}- {\pi }_{{\tilde{A }}_{Si}}^{2})}^{{w}_{i}}\right]}^\frac{1}{2}\right\}.\end{aligned}$$

#### Definition 5

For the spherical weighted geometric mean (SWGM) with respect to, $w=({w}_{1}, {w}_{2}..., {w}_{n})$*,* ${w}_{i} \in [\mathrm{0,1}]$ and ${\sum }_{i=1}^{n}{w}_{i}= 1$, the SWGM is calculated as follows:

17 $$\begin{aligned} & {SWGM}_{w}{(\tilde{A }}_{S1},\dots , {\tilde{A }}_{Sn}) = {\tilde{A }}_{S1}^{{w}_{1}}+{\tilde{A }}_{S2}^{{w}_{2}}+...+{\tilde{A }}_{Sn}^{{w}_{n}}\\ & \quad =\prod\limits_{i=1}^{n}{\mu }_{{\tilde{A }}_{Si}}^{{w}_{i}}, {\left[1-{\prod\limits_{i=1}^{n}(1- {v}_{{\tilde{A }}_{Si}}^{2})}^{{w}_{i}}\right]}^\frac{1}{2},{\left[{\prod\limits_{i=1}^{n}(1- {v}_{{\tilde{A }}_{Si}}^{2})}^{{w}_{i}}- {\prod\limits_{i=1}^{n}(1- {v}_{{\tilde{A }}_{Si}}^{2}- {\pi }_{{\tilde{A }}_{Si}}^{2})}^{{w}_{i}}\right]}^\frac{1}{2}.\end{aligned}$$

In this paper, the SF-AHP model was used to determine the criteria weights of the list of criteria for building the power plant of wind offshore with a case study in Vietnam. The SF-AHP model has five steps, which are described as follows.

Step 1: A hierarchical decision tree is divided into three levels, including the research goal (level 1), list of criteria $C=\{{C}_{1}, {C}_{2},...{C}_{n}\}$(level 2), and location alternatives $A=\{{A}_{1},{A}_{2 },...{A}_{m }\}$ ($within m\ge 2$).

Step 2: Pairwise comparison matrices are performed regarding linguistic terms, as shown in Table 3. The score indices (SI) are determined by Eqs. (18) and (19):

18 $$SI=\sqrt{\left|100* [{({\mu }_{{\tilde{A }}_{S}}-{\pi }_{{\tilde{A }}_{S}})}^{2}-({{v}_{{\tilde{A }}_{S}}-{\pi }_{{\tilde{A }}_{S}})}^{2}]\right|}$$

for the AMI, VHI, HI, SMI, and EI.

19 $$\frac{1}{SI}= \frac{1}{\sqrt{\left|100* [{({\mu }_{{\tilde{A }}_{S}}-{\pi }_{{\tilde{A }}_{S}})}^{2}-({{v}_{{\tilde{A }}_{S}}-{\pi }_{{\tilde{A }}_{S}})}^{2}]\right|}}$$

for the EI, SLI, LI, VLI, and ALI.

**Table 3:** SF-AHP linguistic terms used for pairwise comparisons.

Linguistics terms	Symbol	Fuzzy number $(\mu ,v,\pi )$	Score index (SI)
Absolutely more importance	AMI	(0.9, 0.1, 0.0)	9
Very high importance	VHI	(0.8, 0.2, 0.1)	7
High importance	HI	(0.7, 0.3, 0.2)	5
Slightly more importance	SMI	(0.6, 0.4, 0.3)	3
Equally importance	EI	(0.5, 0.4, 0.4)	1
Slightly low importance	SLI	(0.4, 0.6, 0.3)	1/3
Low importance	LI	(0.3, 0.7, 0.2)	1/5
Very low importance	VLI	(0.2, 0.8, 0.1)	1/7
Absolutely low importance	ALI	(0.1, 0.9, 0.0)	1/9

Step 3: A consistency check is required for pairwise comparison matrices by the consistency ratio (CR), where the CR must be less than 10%.

Step 4: Compute the criterion and alternative spherical fuzzy weights. Determine the weight of each alternative using the SWAM operator using Eq. (20):

20 $$\begin{aligned} & {SWAM}_{w}\left({\tilde{A }}_{S1},\dots , {\tilde{A }}_{Sn}\right)={w}_{1}{\tilde{A }}_{S1}+{w}_{2}{\tilde{A }}_{S2}+...+{w}_{n}{\tilde{A }}_{Sn} \\&\quad =\left\langle {\left[1-{\prod\limits_{i=1}^{n}(1- {\mu }_{{\tilde{A }}_{Si}}^{2})}^{{w}_{i}}\right]}^\frac{1}{2}, \prod\limits_{i=1}^{n}{v}_{{\tilde{A }}_{Si}}^{{w}_{i}},{\left[{\prod\limits_{i=1}^{n}(1- {\mu }_{{\tilde{A }}_{Si}}^{2})}^{{w}_{i}}- {\prod\limits_{i=1}^{n}(1- {\mu }_{{\tilde{A }}_{Si}}^{2}- {\pi }_{{\tilde{A }}_{Si}}^{2})}^{{w}_{i}}\right]}^\frac{1}{2}\right\rangle ,\end{aligned}$$

where $$w= 1/n$$.

Step 5: The final ranking orders for the alternatives are estimated using the defuzzification global weights in Eq. (21):

21 $$S\left({\tilde{w }}_{j}^{s}\right)=\sqrt{\left|100* \left[{\left(3{\mu }_{{\tilde{A }}_{S}}-\frac{{\pi }_{{\tilde{A }}_{S}}}{2}\right)}^{2}-{\left(\frac{{v}_{{\tilde{A }}_{S}}}{2}-{\pi }_{{\tilde{A }}_{S}}\right)}^{2}\right]\right|}.$$

Normalize the criteria weights using Eq. (22) and apply the spherical fuzzy multiplication shown in Eq. (23):

22 $${\overline{w} }_{j}^{s}= \frac{S ({\tilde{w }}_{j}^{s})}{{\sum }_{j=1}^{n}S({\tilde{w }}_{j}^{s})},$$

23 $${\tilde{A }}_{{S}_{ij}}={\overline{w} }_{j}^{s}. {\tilde{A }}_{{S}_{i}}=\langle {{(1-(1-{\mu }_{{\tilde{A }}_{S}}^{2})}^{{\overline{w} }_{j}^{s}})}^{1/2}, {v}_{{\tilde{A }}_{S}}^{{\overline{w} }_{j}^{s}}, {({(1-{\mu }_{{\tilde{A }}_{S}}^{2})}^{{\overline{w} }_{j}^{s}}-{(1- {\mu }_{{\tilde{A }}_{S}}^{2}-{\pi }_{{\tilde{A }}_{S}}^{2})}^{{\overline{w} }_{j}^{s}})}^{1/2} \rangle ,\forall i.$$

The final SF-AHP score ($\tilde{F }$) for each alternative ${A}_{i}$ is obtained by carrying out spherical fuzzy arithmetic addition over each global preference weight, as given in Eq. (24):

$$\tilde{F }=\sum_{j=1}^{n}{\tilde{A }}_{{S}_{ij}} = {\tilde{A }}_{{S}_{i1}}\oplus {\tilde{A }}_{{S}_{i2}}\dots \oplus {\tilde{A }}_{{S}_{in}},\forall i$$

24 $$\mathrm{i}.\mathrm{e}.,{\tilde{A }}_{{S}_{11}}\oplus {\tilde{A }}_{{S}_{12}} = \langle {({\mu }_{{\tilde{A }}_{{S}_{11}}}^{2}+ {\mu }_{{\tilde{A }}_{{S}_{12}}}^{2}-{\mu }_{{\tilde{A }}_{{S}_{11}}}^{2}{\mu }_{{\tilde{A }}_{{S}_{12}}}^{2})}^{1/2}, {v}_{{\tilde{A }}_{{S}_{11}}}{v}_{{\tilde{A }}_{{S}_{12}}},({(1-{\mu }_{{\tilde{A }}_{{S}_{12}}}^{2}){\pi }_{{\tilde{A }}_{{S}_{11}} }^{2}+(1-{\mu }_{{\tilde{A }}_{{S}_{11}}}^{2}){\pi }_{{\tilde{A }}_{{S}_{12}} }^{2}-{\pi }_{{\tilde{A }}_{{S}_{11}} }^{2}{\pi }_{{\tilde{A }}_{{S}_{12}} }^{2})}^{1/2}\rangle$$

The second way to follow is to continue without defuzzification. In this case, spherical fuzzy global preference weights are calculated using Eq. (25):

$$\prod_{j=1}^{n}{\tilde{A }}_{{S}_{ij}}= {\tilde{A }}_{{S}_{i1}} \otimes {\tilde{A }}_{{S}_{i2}}\dots \otimes {\tilde{A }}_{{S}_{in}},\forall i$$

25 $$\mathrm{i}.\mathrm{e}.,{\tilde{A }}_{{S}_{11}} \otimes {\tilde{A }}_{{S}_{12}} = \langle {({\mu }_{{\tilde{A }}_{{S}_{11}}} {\mu }_{{\tilde{A }}_{{S}_{12}}}, ({v}_{{\tilde{A }}_{{S}_{11}}}^{2}+{v}_{{\tilde{A }}_{{S}_{12}}}^{2}-{v}_{{\tilde{A }}_{{S}_{11}}}^{2}{v}_{{\tilde{A }}_{{S}_{12}}}^{2})}^\frac{1}{2}, ({(1-{v}_{{\tilde{A }}_{{S}_{12}}}^{2}){\pi }_{{\tilde{A }}_{{S}_{11}} }^{2}+ (1-{v}_{{\tilde{A }}_{{S}_{11}}}^{2}){\pi }_{{\tilde{A }}_{{S}_{12}} }^{2}-{\pi }_{{\tilde{A }}_{{S}_{11}} }^{2}{\pi }_{{\tilde{A }}_{{S}_{12}} }^{2})}^\frac{1}{2}\rangle .$$

Sort the alternative according to their defuzzified final ratings. The highest value denotes the optimal option.

### Weighted aggregated sum product assessment (WASPAS)

The WASPAS method was proposed in 2012^45^ and is the combination of the weighted product model (WPM) and weighted sum model (WSM); the procedure is explained as follows:

Step 1: A decision matrix is constructed $X={\left[{x}_{ij}\right]}_{m \times n}$, where ${x}_{ij}$ is the performance of the *i*th alternative to the *j*th criterion, $m$ is the number of alternatives and $n$ is the number of criteria.

Step 2: Eqs. (26) and (27) are used to normalize the decision matrix:

For maximizing criteria (benefit):

26 $${\overline{X}}_{ij}=\frac{{x}_{ij}}{{max}_{i}{x}_{ij}}, such \; that \; i=1, 2,\dots ,m;j=1, 2,\dots ,n.$$

For minimizing criteria (nonbenefit):

27 $${\overline{X}}_{ij}=\frac{{min}_{i}{x}_{ij}}{{x}_{ij}}, such \; that \; i=1, 2,\dots ,m;j=1, 2,\dots ,n.$$

Step 3: Eq. (28) is used to calculate the relative importance of the alternative using the weighted sum model (WSM):

28 $${Q}_{i}^{(1)}=\sum_{j=1}^{n}{\overline{X}}_{ij}{w}_{j}, such \; that \; i=1, 2, \dots ,m,$$

where ${w}_{j}$ is the weight (relative importance) of the *j*th criterion.

Step 4: The relative importance of the alternative is then calculated using the weighted product model (WPM), as shown in Eq. (29):

29 $${Q}_{i}^{(2)}=\prod_{j=1}^{n}{\left({\overline{X}}_{ij}\right)}^{{w}_{j}}, such \; that \; i=1, 2, \dots ,m,$$

where ${w}_{j}$ is the weight (relative importance) of the *j*th criterion. In this paper, ${w}_{j}$ is obtained from SF-AHP model.

Step 5: The integrated utility function of the WASPAS model is calculated using Eq. (30):

30 $${Q}_{i}=\lambda {Q}_{i}^{(1)}+\left(1-\lambda \right){Q}_{i}^{\left(2\right)}=\lambda \sum_{j=1}^{n}{\overline{X}}_{ij}{w}_{j}+\left(1-\lambda \right)\prod_{j=1}^{n}{\left({\overline{X}}_{ij}\right)}^{{w}_{j}},\lambda =0,\dots ,1.$$

The value of $\lambda$ (coefficient value or threshold value of the WASPAS model) is determined using Eq. (31):

31 $$\lambda =\frac{\sum_{i=1}^{m}{Q}_{i}^{(2)}}{\sum_{i=1}^{m}{Q}_{i}^{(1)}+\sum_{i=1}^{m}{Q}_{i}^{(2)}}.$$

## Results analysis

### A case study in Vietnam

With 3000 km of coastline and winds ranging from 5.5 to 7.3 m/s at 100 m, Vietnam has an exceptional natural wind potential (not accounting for seasonal variability). Offshore, the best chance for large-scale wind power generation exists. According to the World Bank, Vietnam's offshore wind potential could be as high as 500 GW. Acorrding to the Danish Energy Agency (DEA) and the Vietnamese Electricity and Renewable Energy Authority^61^, Vietnam’s average wind speeds are 7.2 m/s at 100 m, and more than 39% of the total area of Vietnam is estimated to have an average annual wind speed greater than 6 m/s at an altitude of 65 m.

In this section, the proposed aggregated framework is executed to identify the most suitable OWPS construction locations. A panel of 15 experts with at least ten years of professional experience in energy-related fields was invited to determine potential alternatives and essential criteria for the evaluation to increase the objectivity of the results as much as possible. Some of them are professionals who are experts with a background in the field of energy and projects for the construction and assessment of wind power plants. The others are specialized experts in various fields—such as electrical engineering, hydrology, social science, construction, and the environment—associated with site selection for OWPS. The committee also consulted with the Vietnam Institute of Energy and relevant contributors interested in gathering information about building wind towers, wind resources, and marine wind energy planning. Many vital considerations proposed by governmental and non-governmental organizations were referenced and discussed between experts and operators to filter out potential alternatives for OWPS in Vietnam. Some restrictions linked to environmental and social standards during the selection process can be named, such as ecological protection zones, restricted military areas, oil and gas operations, fishery, shipping, historical and cultural heritage, and many more. After discussions, six locations were selected as potential alternatives: Ba Ria-Vung Tau, Ben Tre, Binh Dinh, Binh Thuan, Ca Mau, Ninh Thuan, and Soc Trang. With good wind resources, these six potential offshore locations are also considered excellent areas among those planned and supported for offshore wind farms announced by the government most recently. Table 4 shows each location’s wind speed and wind density on average at an altitude of 100 m. The evaluation indicator system was constructed through reviews, discussion, literature and finalized by the invited experts. As a result, 15 criteria were selected, as defined in Table 5.

**Table 4:** List of potential offshore wind locations in Vietnam.

DMU	Location	Wind speed (m/s)	Wind density (W/m^2^)
OWPS-01	Ba Ria—Vung Tau	6.33	235
OWPS-02	Ben Tre	6.24	225
OWPS-03	Binh Dinh	7.87	627
OWPS-04	Binh Thuan	8.18	673
OWPS-05	Ca Mau	5.94	196
OWPS-06	Ninh Thuan	9.30	935
OWPS-07	Soc Trang	6.25	216

**Table 5:** The criteria used in the paper and their definition.

Dimensions	Criteria	Definition
C1. Wind resources	C11. Wind speed and its distribution status	Based on the average annual wind force, the wind and monsoon conditions in places where wind farms are constructed. The wind speed computation would be based on long-term representative wind speed fluctuations in the area and investigated on site
	C12. Effective wind hours	Refers to accumulative hours of practical usage of wind power per year (h)
C2. Environmental impact	C21. Nautical life coordination	The distance between OWPS and marine life migration determines the degree of coordination with sea area planning for marine life. Depending on the geographical context, the generator machine's selection and installation would disrupt the original seabed during construction. At the same time, the turbine would generate noise pollution throughout its rotation, resulting in low-frequency sound waves that would be harmful to marine species engaged in predation or migratory behaviors
	C22. Nautical environmental influence	The potential for OWPS to degrade the quality of the marine ecology and biodiversity
C3. Construction and maintenance conditions	C31. Seawater depth	The suitability of OWPS building also takes into account the depth of the sea, the distance from the coast, and the width of the shore
	C32. Undersea geological conditions	This criterion assesses regional geological conditions and construction stability based on acquired data and geological prospecting
	C33. Marine conditions	Characteristics of the sea area like waves, tidal current, temperature, storm surge, sea ice, sea bed movement, and erosion must be considered when evaluating the hazard of complex hydrological conditions on project safety
C4. Societal impact	C41. Employment	The related manufacturing and service industries would grow with the project's development, and various possible job incentives would surface one after another when determining a construction location for OWPS. As a result, it is required to use employment to assess the impact, such as which station sites affect salary, relevant industries, etc. Knowing the position of the staff, the work environment, and other factors might have an impact on employment
	C42. Policy planning	The central government's and local governments' support and promote wind farm construction; this criterion also considers if necessary legislation and policies have been implemented to encourage offshore wind projects
C5. Conditions onshore	C51. Distance from the power load center	The distance between the area and the electrical load center is the distance over which electricity is transmitted from the power station to the shore (submarine cable)
	C52. Electrical transmission and distribution system	The electrical system's capacity to meet future power supply requirements (e.g., substation, electrical grid)
	C53. Traffic condition	Examines the ease with which huge equipment can be transported along the shore (e.g., highway, railway, bridge, airport, dock)
C6. Economic impact	C61. Cost-to-benefit ratio	Typically, the offshore wind power profit and loss balance is utilized in estimations
	C62. Construction, operation, and maintenance costs	This criterion shows the total cost of the OWPS projects, from conception to completion and delivery in its final form, and all operating and maintenance expenditures in the surrounding area after the offshore wind farm is fully operational
	C63. Provincial financial subsidies	Relates to the subsidies promoted by the local government finance

### Results of the SF-AHP model

In this stage, an example of the following calculation of the six main criteria presents the SF-AHP procedure: wind resources (C1), environmental impact (C2), construction and maintenance conditions (C3), societal impact (C4), onshore conditions (C5), and economic impact (C6). The same procedures were applied to calculate the relative importance of the potential wind locations concerning the predetermined 15 criteria. The linguistic terminology is used to express the opinion of specialists in evaluating one criterion relative to another, as shown in Table 3. This evaluation process is based on the experts’ experience to implement this step with the local conditions and offshore characteristics. Next, the pairwise comparison matrix using linguistic terms, the nonfuzzy comparison matrix, and the normalized comparison matrix of the SF-AHP model are presented in Tables 6, 7 and 8. The consistency verification of the pairwise comparison matrices was computed as follows:

**Table 6:** The pairwise comparison matrix of the SF-AHP model.

Criteria	Left criteria is greater					Right criteria is greater				Criteria
	AMI	VHI	HI	SMI	EI	SLI	LI	VLI	ALI	
C1		6	2	6	1					C2
C1		5	3	4	3					C3
C1						6	6	2	1	C4
C1	5	4	4	2						C5
C1		1	3	2	4	5				C6
C2			1	3	5	6				C3
C2						1	4	4	6	C4
C2			6	4	4	1				C5
C2					2	4	4	5		C6
C3						1	5	4	5	C4
C3			5	5	4	1				C5
C3					2	3	4	6		C6
C4	1	3	5	6						C5
C4		6	5	3	1					C6
C5				1	3	2	4	5		C6

**Table 7:** The nonfuzzy comparison matrix of the SF-AHP model.

Criteria	C1	C2	C3	C4	C5	C6
C1	1.000	4.189	3.538	0.226	6.215	1.261
C2	0.239	1.000	0.894	0.150	2.371	0.254
C3	0.283	1.119	1.000	0.156	2.292	0.240
C4	4.433	6.687	6.430	1.000	4.534	4.639
C5	0.161	0.422	0.436	0.221	1.000	0.316
C6	0.793	3.938	4.167	0.216	3.162	1.000
SUM	6.909	17.355	16.465	1.967	19.574	7.710

**Table 8:** The normalized comparison matrix of the SF-AHP model.

Criteria	C1	C2	C3	C4	C5	C6	MEAN	WSV	CV
C1	0.145	0.241	0.215	0.115	0.318	0.164	0.199	1.296	6.496
C2	0.035	0.058	0.054	0.076	0.121	0.033	0.063	0.390	6.212
C3	0.041	0.064	0.061	0.079	0.117	0.031	0.066	0.410	6.251
C4	0.642	0.385	0.391	0.508	0.232	0.602	0.460	3.166	6.883
C5	0.023	0.024	0.026	0.112	0.051	0.041	0.046	0.287	6.197
C6	0.115	0.227	0.253	0.110	0.162	0.130	0.166	1.090	6.571

$${C}_{12}=\frac{{SI}_{{C}_{12}}}{{SUM}_{{C}_{2}}}=\frac{4.189}{17.355}=0.241,$$

$${MEAN}_{{C}_{1}}=\frac{0.145+0.241+0.215+0.115+0.318+0.164}{6}=0.199,$$

$$WSV=\left[\begin{array}{ccccc}1.000& 4.189& 3.538& 0.226& \begin{array}{cc}6.215& 1.261\end{array}\\ 0.239& 1.000& 0.894& 0.150& \begin{array}{cc}2.371& 0.254\end{array}\\ 0.283& 1.119& 1.000& 0.156& \begin{array}{cc}2.292& 0.240\end{array}\\ 4.433& 6.687& 6.430& 1.000& \begin{array}{cc}4.534& 4.639\end{array}\\ \begin{array}{c}0.161\\ 0.793\end{array}& \begin{array}{c}0.422\\ 3.938\end{array}& \begin{array}{c}0.436\\ 4.167\end{array}& \begin{array}{c}0.221\\ 0.216\end{array}& \begin{array}{c}\begin{array}{cc}1.000& 0.316\end{array}\\ \begin{array}{cc}3.162& 1.000\end{array}\end{array}\end{array}\right]\times \left[\begin{array}{c}0.199\\ 0.063\\ 0.066\\ 0.460\\ \begin{array}{c}0.046\\ 0.166\end{array}\end{array}\right]=\left[\begin{array}{c}1.296\\ 0.390\\ 0.410\\ 3.166\\ \begin{array}{c}0.287\\ 1.090\end{array}\end{array}\right],$$

$$CV={\left[\begin{array}{c}1.296\\ 0.390\\ 0.410\\ 3.166\\ \begin{array}{c}0.287\\ 1.090\end{array}\end{array}\right]}/{\left[\begin{array}{c}0.199\\ 0.063\\ 0.066\\ 0.460\\ \begin{array}{c}0.046\\ 0.166\end{array}\end{array}\right]}=\left[\begin{array}{c}6.496\\ 6.212\\ 6.251\\ 6.883\\ \begin{array}{c}6.197\\ 6.571\end{array}\end{array}\right].$$

With the six main criteria ($n=6$), the largest eigenvector (${\uplambda }_{max}$) was calculated to identify the consistency index (*CI*), the random index (*RI*), and consistency ratio (*CR*) as follows:

$${\uplambda }_{max}=\frac{6.496+6.212+6.251+6.883+6.197+6.571}{6}=6.435,$$

$$CI=\frac{{\uplambda }_{max}-n}{n-1}=\frac{6.435-6}{6-1}=0.087,$$

where $n=6$, $RI=1.24$, and the *CR* value is calculated as follows:

$$CR=\frac{CI}{RI}=\frac{0.087}{1.24}=0.070.$$

As shown in $CR=0.070<0.1$, the pairwise comparison matrix was consistent, and the result was satisfactory.

Following that, the integrated spherical fuzzy comparison matrix is calculated in Table 9. Then, the obtained spherical fuzzy weights of each criterion were calculated and are shown in Table 10. For explanation, the following calculation was presented for the spherical fuzzy weights of criteria C1 with $(\mu ,v,\pi )=(0.610, 0.388, 0.274)$, as follows:

**Table 9:** The integrated spherical fuzzy comparison matrix.

	C1			C2			C3			C4			C5			C6		
	$\mu$	$v$	$\pi$	$\mu$	$v$	$\pi$	$\mu$	$v$	$\pi$	$\mu$	$v$	$\pi$	$\mu$	$v$	$\pi$	$\mu$	$v$	$\pi$
C1	0.500	0.400	0.400	0.679	0.323	0.245	0.657	0.328	0.270	0.296	0.708	0.215	0.773	0.246	0.166	0.525	0.462	0.312
C2	0.292	0.708	0.217	0.500	0.400	0.400	0.485	0.492	0.331	0.485	0.492	0.331	0.592	0.385	0.304	0.303	0.696	0.221
C3	0.300	0.692	0.237	0.461	0.509	0.332	0.500	0.400	0.400	0.190	0.813	0.122	0.586	0.391	0.309	0.289	0.710	0.208
C4	0.681	0.328	0.236	0.794	0.224	0.145	0.781	0.236	0.154	0.500	0.400	0.400	0.687	0.323	0.233	0.700	0.300	0.222
C5	0.194	0.810	0.129	0.366	0.616	0.289	0.373	0.608	0.296	0.288	0.716	0.209	0.500	0.400	0.400	0.316	0.682	0.227
C6	0.413	0.572	0.297	0.652	0.341	0.257	0.665	0.329	0.247	0.275	0.724	0.197	0.618	0.372	0.270	0.500	0.400	0.400

**Table 10:** The spherical weights from the SF-AHP model.

	SF-AHP weights			Calculations to obtain crisp weights	Crisp weights
	$\mu$	$v$	$\pi$	$S\left({\tilde{w }}_{j}^{s}\right)$	${\overline{w} }_{j}^{s}$
C1	0.610	0.388	0.274	16.915	0.200
C2	0.462	0.514	0.315	12.268	0.145
C3	0.420	0.563	0.296	11.124	0.131
C4	0.707	0.296	0.232	20.039	0.237
C5	0.356	0.624	0.282	9.259	0.109
C6	0.551	0.437	0.288	15.072	0.178

$$\begin{aligned} {\mu }_{{C1}}& ={{\left[1-{\prod\limits_{i=1}^{n}(1- {\mu }_{{A}_{Si}}^{2})}^{{w}_{i}}\right]}^{1/2}}\\ &={\left[1-{\left(1-{0.500}^{2}\right)}^\frac{1}{6}*{\left(1-{0.679}^{2}\right)}^\frac{1}{6}*{\left(1-{0.657}^{2}\right)}^\frac{1}{6}{*\left(1-{0.296}^{2}\right)}^\frac{1}{6}*{\left(1-{0.773}^{2}\right)}^\frac{1}{6}*{\left(1-{0.525}^{2}\right)}^\frac{1}{6}\right]}^{1/2}\\ &=0.610,\end{aligned}$$

$${v}_{C1}=\prod\limits_{i=1}^{n}{v}_{{A}_{Si}}^{{w}_{i}}={{0.400}^\frac{1}{6}*0.323}^\frac{1}{6}*{0.328}^\frac{1}{6}*{0.708}^\frac{1}{6}*{0.246}^\frac{1}{6}*{0.462}^\frac{1}{6}=0.388,$$

$$\begin{aligned}{\pi }_{C1}&={\left[{\prod\limits_{i=1}^{n}(1- {\mu }_{{A}_{Si}}^{2})}^{{w}_{i}}- {\prod\limits_{i=1}^{n}(1- {\mu }_{{A}_{Si}}^{2}- {\pi }_{{A}_{Si}}^{2})}^{{w}_{i}}\right]}^{1/2}\\ & ={\left[{\left(1-{0.500}^{2}\right)}^\frac{1}{6}*{\left(1-{0.679}^{2}\right)}^\frac{1}{6}*{\left(1-{0.657}^{2}\right)}^\frac{1}{6}{*\left(1-{0.296}^{2}\right)}^\frac{1}{6}*{\left(1-{0.773}^{2}\right)}^\frac{1}{6}\right.}\\&\quad{\left.*{\left(1-{0.525}^{2}\right)}^\frac{1}{6}-{\left(1-{0.500}^{2}-{0.400}^{2}\right)}^\frac{1}{6}*{\left(1-{0.679}^{2}-{0.245}^{2}\right)}^\frac{1}{6}*{\left(1-{0.657}^{2}-{0.270}^{2}\right)}^\frac{1}{6}\right.}\\&\quad{\left.{*\left(1-{0.296}^{2}-{0.215}^{2}\right)}^\frac{1}{6}*{\left(1-{0.773}^{2}-{0.166}^{2}\right)}^\frac{1}{6}*{\left(1-{0.525}^{2}-{0.312}^{2}\right)}^\frac{1}{6}\right]}^{1/2}=0.274,\end{aligned}$$

$$\begin{aligned}S \left({\tilde{w }}_{C1}^{s}\right)&=\sqrt{\left|100*\left[{\left(3{\mu }_{{\overline{A} }_{s }}-\frac{{\pi }_{{\overline{A} }_{s }}}{2}\right)}^{2}-{\left(\frac{{v}_{{\overline{A} }_{s }}}{2}-{\pi }_{{\overline{A} }_{s }}\right)}^{2}\right]\right|}\\ &=\sqrt{\left|100*\left[{\left(3*0.610-\frac{0.274}{2}\right)}^{2}-{\left(\frac{0.388}{2}-0.274\right)}^{2}\right]\right|}=16.915,\end{aligned}$$

$${\overline{w} }_{C1}^{s}=\frac{S ({\tilde{w }}_{j}^{s})}{{\sum }_{\mathrm{j}=1}^{n}S({\tilde{w }}_{j}^{s})}=\frac{16.915}{16.915+12.268+11.124+20.039+9.259+15.072}=0.200.$$

The SF-AHP weights of the six main criteria consist of three parameters: the membership degree ($$\mu )$$, nonmembership degree ($$v)$$, and hesitancy degree ($$\pi$$) of the element $$x\in X$$. The crisp weights of the six main criteria were calculated based on the abovementioned calculation. The criteria of societal impact (C4) with a value of 0.237, wind resources (C1) with a value of 0.200, and economic impact (C6) with a value of 0.178 are determined to be the most critical criteria in the stage of the SF-AHP model. Consequently, the same steps are applied to calculate the significance level of other criteria of the first stage of the paper. The integrated spherical fuzzy comparison matrix of all criteria is presented in Table A1 (Appendix A).

**Figure 4:**
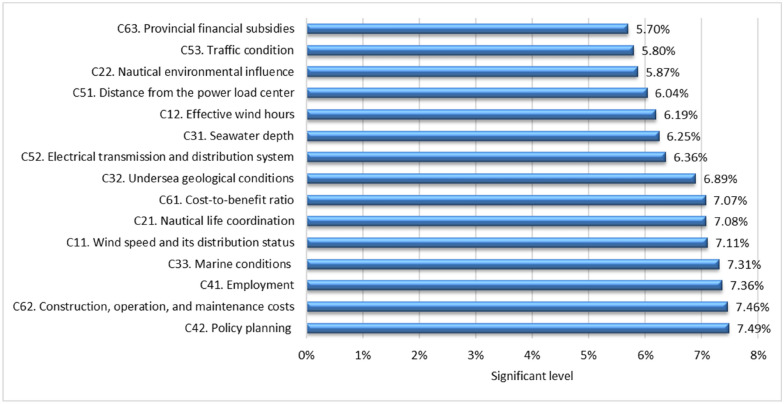
The influence level of criteria of the SF-AHP model.

Table 11 displays the spherical fuzzy weights and crisp weights of the SF-AHP model. The geometrical mean, defuzzification, and normalization procedures are used to calculate the influence level of each criterion. For example, the spherical fuzzy weights of the criteria wind speed and its distribution status (C11) have a membership degree ($$\mu )$$ at 0.506, nonmembership degree ($$v)$$ at 0.473, and hesitancy degree ($$\pi$$) at 0.311. Similar to the procedure, the spherical fuzzy weights of the criteria effective wind hours (C12) have membership degrees ($$\mu )$$, nonmembership degrees ($$v)$$, and hesitancy degrees ($$\pi$$) of 0.447, 0.538, and 0.310, respectively. The significance level of the criteria of the SF-AHP model is shown in Fig. 4. The results show that the five most significant criteria for determining the OWPS sites are policy planning (C42); construction, operation, and maintenance costs (C62); employment (C41), marine conditions (C33), and wind speed and its distribution status (C11), with significance levels of 7.49%, 7.46%, 7.36%, 7.31, and 7.11%, respectively. Meanwhile, provincial financial subsidies (C63) are specified as the least significant criterion, with a value of 7.70%. The findings suggest that decision-makers should pay more attention to “C42”, “C62”, “C41”, “C33”, and “C11” than other criteria.

**Table 11:** Spherical fuzzy weights and crisp weights of the SF-AHP model.

Criteria	Geometric mean			Spherical fuzzy weights			Crisp weights
	$\mu$	$v$	$\pi$	$\mu$	$v$	$\pi$	
C11. Wind speed and its distribution status	0.744	0.473	0.097	0.506	0.473	0.311	0.071
C12. Effective wind hours	0.800	0.538	0.096	0.447	0.538	0.310	0.062
C21. Nautical life coordination	0.740	0.473	0.115	0.510	0.473	0.339	0.071
C22. Nautical environmental influence	0.819	0.566	0.092	0.425	0.566	0.303	0.059
C31. Seawater depth	0.797	0.535	0.097	0.451	0.535	0.311	0.062
C32. Undersea geological conditions	0.756	0.496	0.102	0.494	0.496	0.320	0.069
C33. Marine conditions	0.723	0.457	0.122	0.526	0.457	0.350	0.073
C41. Employment	0.720	0.451	0.119	0.529	0.451	0.345	0.074
C42. Policy planning	0.717	0.451	0.104	0.532	0.451	0.322	0.075
C51. Distance from the power load center	0.812	0.563	0.083	0.433	0.563	0.289	0.060
C52. Electrical transmission and distribution system	0.791	0.533	0.093	0.457	0.533	0.305	0.064
C53. Traffic condition	0.823	0.570	0.093	0.421	0.570	0.305	0.058
C61. Cost-to-benefit ratio	0.743	0.468	0.108	0.507	0.468	0.328	0.071
C62. Construction, operation, and maintenance costs	0.719	0.457	0.101	0.530	0.457	0.318	0.075
C63. Provincial financial subsidies	0.829	0.581	0.088	0.413	0.581	0.297	0.057

### Results of the WASPAS model

**Figure 5:**
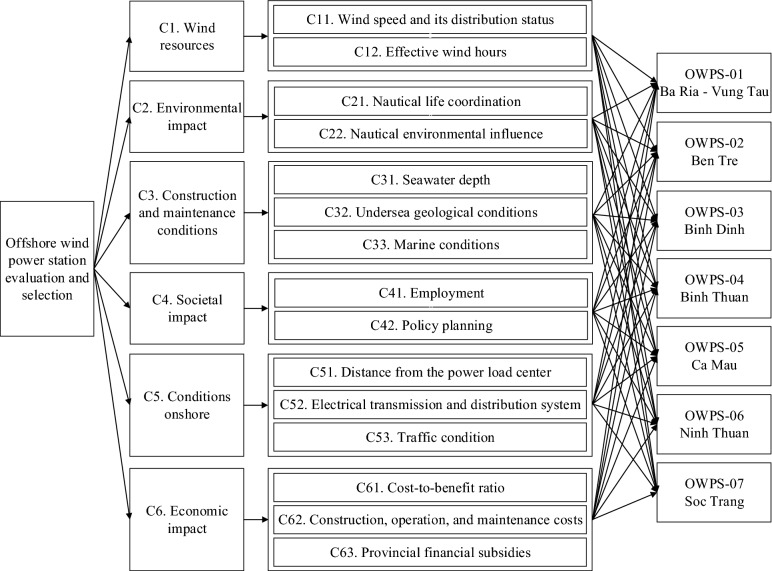
The decision tree of the evaluation process.

In the second stage, this paper deployed the SF-AHP weights to combine with the WASPAS model for ranking the potential wind plants, which are Ba Ria—Vung Tau (OWPS-01), Ben Tre (OWPS-02), Binh Dinh (OWPS-03), Binh Thuan (OWPS-04), Ca Mau (OWPS-05), Ninh Thuan (OWPS-06), and Soc Trang (OWPS-07). The decision hierarchy tree for the goal of OWPS site selection is depicted in Fig. 5.

**Figure 6:**
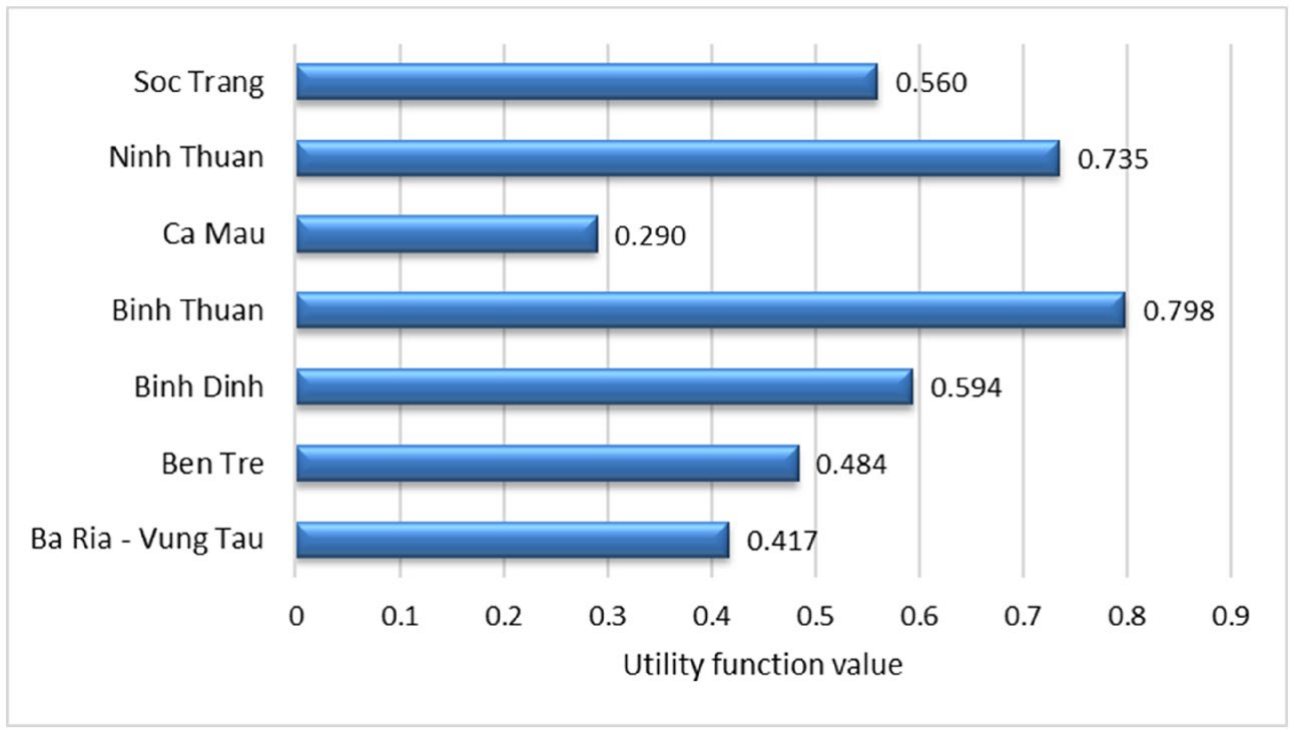
Location ranking of the WASPAS model.

The weighted normalized matrix for the WSM and weighted normalized matrix for the WPM are displayed in Tables A2 and A3, respectively. The WASPAS model ranks the alternative based on the integrated simple additive weighting and exponentially weighted product model to obtain a compromise solution. Table 12 presents the integrated utility function ${Q}_{i}$ of the WASPAS model, which is calculated using the weighted sum model ${Q}_{i}^{(1)}$ (WSM) and the weighted product model ${Q}_{i}^{(2)}$ (WSM). The results show that the top three offshore wind locations are Binh Thuan (OWPS-04), Ninh Thuan (OWPS-06), and Binh Dinh (OWPS-03), ranking in the first, second, and third positions with scores of 0.798, 0.735, and 0.594, respectively. Figure 6 displays the final location ranking from the WASPAS model.

**Table 12:** The integrated utility function of the WASPAS model.

DMU	Location	${Q}_{i}^{(1)}$	${Q}_{i}^{(2)}$	${Q}_{i}$	Ranking
OWPS-01	Ba Ria—Vung Tau	0.426	0.408	0.417	6
OWPS-02	Ben Tre	0.494	0.474	0.484	5
OWPS-03	Binh Dinh	0.615	0.572	0.594	3
OWPS-04	Binh Thuan	0.842	0.754	0.798	1
OWPS-05	Ca Mau	0.329	0.252	0.290	7
OWPS-06	Ninh Thuan	0.766	0.704	0.735	2
OWPS-07	Soc Trang	0.576	0.544	0.560	4

## Results validation

To confirm the reliability and accuracy of the results and to check the robustness of the proposed approach, the following validation methods are compared with existing plant locations, sensitivity analysis, and MCDM technique comparisons.

### Comparison with the existing locations

The final ranking of locations of their sustainability for OWPS construction is validated in Table 13, which displays the provinces’ total capacity of normal status OWPS projects in Vietnam as of 2021^3^. Most projects are now concentrated in Binh Thuan (15,800 MWp), Ninh Thuan (4280 MWp), and Binh Dinh (2900 MWp). Offshore wind energy can also be expanded throughout the country toward a clean energy development pathway; however, given the results, the authors recommend further analysis of these areas as they are very promising.

**Table 13:** OWPS projects in Vietnam as of 2021.

DMU	Location	Capacity (MWp)
OWPS-01	Ba Ria—Vung Tau	1000
OWPS-02	Ben Tre	900
OWPS-03	Binh Dinh	2900
OWPS-04	Binh Thuan	15,800
OWPS-05	Ca Mau	350
OWPS-06	Ninh Thuan	4280
OWPS-07	Soc Trang	800

### Sensitivity analysis

To demonstrate the robustness and stability of the proposed MCDM model, a sensitivity analysis is conducted for the parameters including the preference coefficient and the index weights^62^.

**Figure 7:**
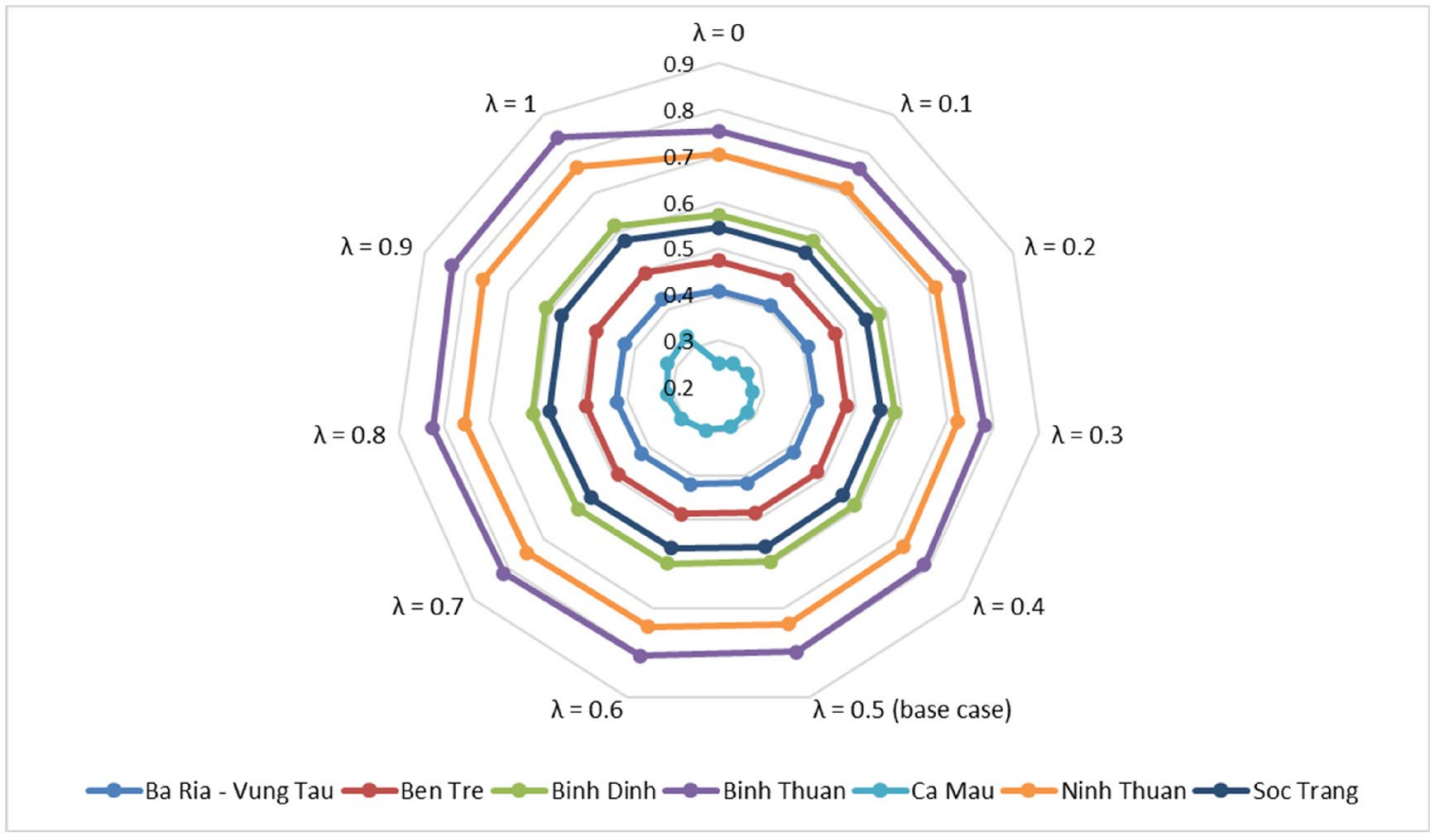
Sensitivity analysis of the threshold value of the WASPAS model.

First, a sensitivity analysis of the preference coefficient (i.e., the threshold value of the WASPAS model, $\lambda$) is conducted to validate the ranking order^63^. In a previous relevant study, the value of λ was considered to be 0.5 ($\lambda =0.5$) for base case analysis. However, this setting does not reflect the actual scenario in which various decision-makers have different preferences. Hence, in this paper, the preference coefficient of the WASPAS model fluctuates in the range of $(\lambda =0, 0.1,\dots , 1)$, as shown in Table 14. The change result is visualized in Fig. 7. The ranking result shows that the optimal location for building the offshore wind station is always the same when changing the values of coefficient preference ($\lambda$) from 0 to 1. It can be concluded that Binh Thuan (OWPS-04) is consistently the optimal location to take over. Following that, Ninh Thuan (OWPS-06) and Binh Dinh (OWPS-03) are also ranked second and third positions, which are also more suitable alternatives among other candidates. The reliability and correctness of the proposed model are demonstrated. Decision-maker psychology should be considered when making decisions in determining the optimal wind plant location from multiple alternatives.

**Table 14:** The fluctuation threshold value of the WASPAS model.

DMU	Location	Coefficient values ($\lambda$)										
		0	0.1	0.2	0.3	0.4	0.5	0.6	0.7	0.8	0.9	1
OWPS-01	Ba Ria—Vung Tau	0.408	0.410	0.412	0.414	0.415	0.417	0.419	0.421	0.422	0.424	0.426
OWPS-02	Ben Tre	0.474	0.476	0.478	0.480	0.482	0.484	0.486	0.488	0.490	0.492	0.494
OWPS-03	Binh Dinh	0.572	0.577	0.581	0.585	0.590	0.594	0.598	0.602	0.607	0.611	0.615
OWPS-04	Binh Thuan	0.754	0.762	0.771	0.780	0.789	0.798	0.806	0.815	0.824	0.833	0.842
OWPS-05	Ca Mau	0.252	0.259	0.267	0.275	0.282	0.290	0.298	0.306	0.313	0.321	0.329
OWPS-06	Ninh Thuan	0.704	0.710	0.717	0.723	0.729	0.735	0.741	0.748	0.754	0.760	0.766
OWPS-07	Soc Trang	0.544	0.547	0.551	0.554	0.557	0.560	0.563	0.567	0.570	0.573	0.576

**Figure 8:**
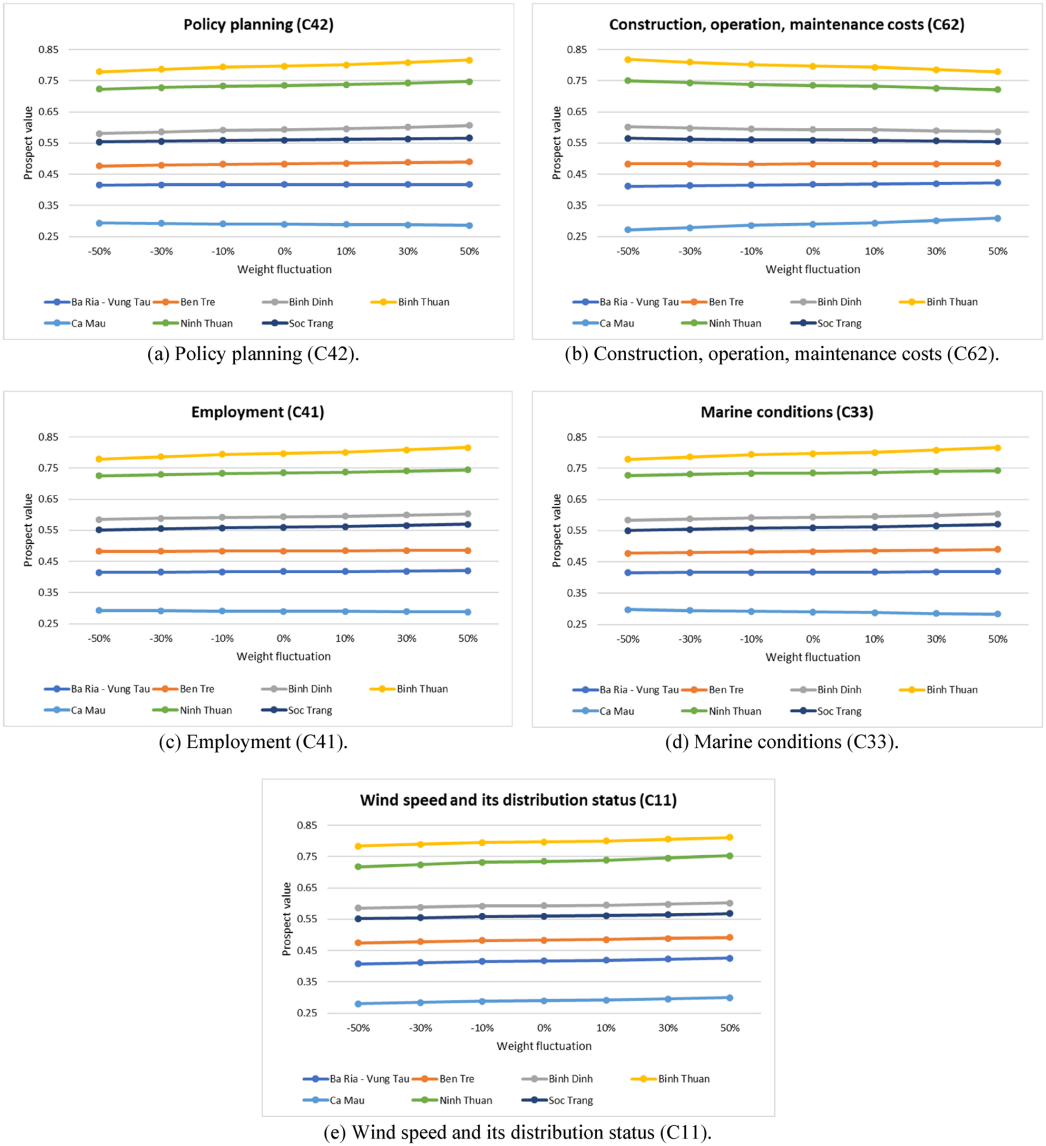
Sensitivity analysis of the five most significant criteria.

Second, a sensitivity analysis of criteria is conducted to investigate the impact of criteria on the ranking of wind plant locations. The top five main criteria are selected to fluctuate their weights from $\pm 10\%, \pm 30\%$ and $\pm 50\%$^64^, which are policy planning (C42); construction, operation, and maintenance costs (C62); employment (C41); marine conditions (C33); and wind speed and its distribution status (C11). In total, there will be 30 scenarios of sensitivity analysis in this case. Figure 8 depicts that the final ranking results of the seven locations are fundamentally stable. The results show that Binh Thuan (OWPS-04) and Ninh Thuan (OWPS-06) are always ranked first and second on $10\%, 30\%, 50\%$ more weight and $10\%, 30\%, 50\%$ less weight than the base case. Generally, the curve is relatively smooth, revealing that the ranking result of the proposed MCDM model of SF-AHP and WASPAS is stable and applicable.

### Comparative analysis

In the MCDM approach, the applicability and rationality of the proposed methods must be proven by comparison with stable and mature methods commonly used in related studies. In this paper, the ranking of wind locations using the integrated SF-AHP and WASPAS models is evaluated by comparison with the Technique for Order of Preference by Similarity to Ideal Solution (TOPSIS), Combined Compromise Solution (CoCoSo), and Evaluation Based on Distance from Average Solution (EDAS). The TOPSIS method is known as the classical MCDM model based on the concept that the selected alternative should have the shortest distance from the positive ideal solution and the farthest distance from the negative ideal solution^65^. The EDAS method could be used most effectively for solving a larger number of complex decision-making problems^66^. Meanwhile, the CoCoSo method has algorithm steps similar to those of the WASPAS method, which was built based on aggregated simple additive weighting and an exponentially weighted product model to obtain a compromise solution^67^.

**Figure 9:**
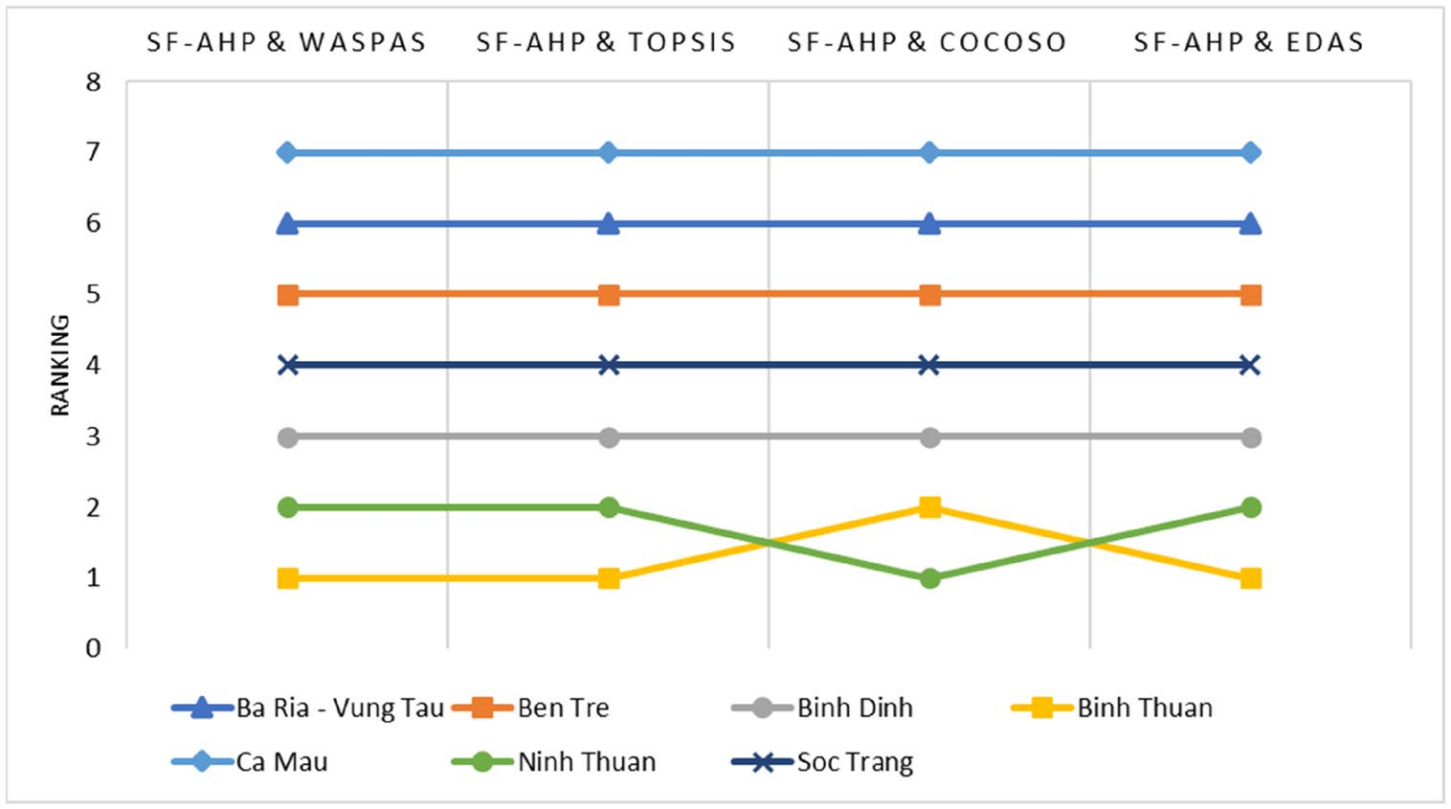
Ranking results of compared methods.

The comparison of four kinds of ranking methods is shown in Table 15 and visualized in Fig. 9. The comparison shows that the ranking of the offshore wind location has given the same result as the model proposed in this paper, which is among integrated models of SF-AHP and WASPAS, SF-AHP and TOPSIS, and SF-AHP and EDAS. The ranking of the SF-AHP and CoCoSo models is slightly different from that of the proposed model. The difference is between Binh Thuan (OWPS-04) and Ninh Thuan (OWPS-06). Hence, the proposed MCDM integrated model is robust, and the obtained result is reliable and can be a useful guideline for decision-makers, investors, or governments in determining the optimal offshore wind plants in Vietnam or related industries.

**Table 15:** The comparison of four kinds of ranking methods.

DMU	Location	SF-AHP WASPAS		SF-AHP TOPSIS		SF-AHP COCOSO		SF-AHP EDAS	
		Value	Ranking	Value	Ranking	Value	Ranking	Value	Ranking
OWPS-01	Ba Ria—Vung Tau	0.417	6	0.347	6	4.240	6	0.290	6
OWPS-02	Ben Tre	0.484	5	0.409	5	4.764	5	0.392	5
OWPS-03	Binh Dinh	0.594	3	0.528	3	5.232	3	0.567	3
OWPS-04	Binh Thuan	0.798	1	0.681	1	5.654	2	0.919	1
OWPS-05	Ca Mau	0.290	7	0.287	7	0.910	7	0.092	7
OWPS-06	Ninh Thuan	0.735	2	0.670	2	5.926	1	0.825	2
OWPS-07	Soc Trang	0.560	4	0.495	4	5.090	4	0.510	4

## Managerial implications

The aim of decreasing global greenhouse gas emissions will depend mainly on developing economies such as Vietnam. Critically, the COVID-19 epidemic has highlighted the vulnerabilities in this fossil fuel-based economy, accelerating the transition faster than before. Pathways toward low carbon development, Vietnam is tapping into its good wind energy potentials. However, up to now, offshore wind development is still a brand-new field in Vietnam. From the proposed evaluation framework, “policy planning” was recognized as the most impactful criteria in developing offshore wind in Vietnam. From the lens of experts and scholars, with a transparent legal framework and suitable support mechanisms, offshore wind can quickly develop and compete on price with other power sources. Accordingly, discussions emphasized the urgency of developing a complete policy framework and suitable support mechanisms for offshore wind power development, whereby, in order to achieve net-zero emissions by 2050. Offshore wind power in Vietnam needs to be carefully prepared in terms of the policy framework, including support mechanisms, licensing procedures, as well as power purchase agreements in accordance with international standards.

In this paper, an effective method for selecting the appropriate locations for offshore wind plant installation has been established. Locations for OWPS construction in Vietnam were examined concerning sustainability criteria; the proposed approach successfully determined optimal alternatives. Binh Thuan, Ninh Thuan, and Binh Dinh have been the best areas for OWPS construction according to the final ranking of WASPAS analysis. A comparison with the existing areas for OWPS and sensitivity analysis of criteria are presented to support the obtained results. Additional comparisons are conducted with other MCDM methods (TOPSIS, CoCoSo, and EDAS). Accordingly, the priority order of the best locations is similar, indicating that the proposed methodology is robust. The applied sensitivity analysis will allow decision-makers to test the observation stability. The study provides a decision support tool that assists authorities and decision-makers in developing suitable and effective planning strategies for OWPS projects. Since the tools used in the study can be applied anywhere in the world, this study can be a helpful guide for other researchers, governments, or private investors. By using the MCDM models, a basis for informed decisions is provided to save costs and resources in the planning phase of offshore wind plants. The proposed model can be replicated for similar site selection problems (e.g. tidal power generation sites, solar panel installation locations, wave energy development, and hybrid energy systems) with appropriate modification of the criteria and alternatives required to meet the actual situations.

## Concluding remarks

This study proposes a new design of a comprehensive MCDM framework combining the SF-AHP and WASPAS methods for the OWPS site selection problem, and a real case study in Vietnam is considered. The problems caused by experts evaluating with ambiguous linguistic terms are overcome by presenting the assessments in the form of the spherical fuzzy environment that does not omit expert information. The proposed framework incorporates sustainability aspects while building on previous offshore wind power assessment studies and experts' knowledge. The most important criteria, such as wind resources, environmental impact, construction and maintenance conditions, societal impact, conditions onshore, and economic impact, were considered. The SF-AHP method in the first stage determines the significance levels of OWPS evaluation criteria. WASPAS is then utilized to rank locations of OWPS. We conducted comparisons with selected other MCDM methods and tested the robustness of the suggested model by completing a sensitive analysis. Although the proposed model can assist decision-makers in selecting a location, different measurement tools are still required. One of the study's limitations is that the evaluation process of site selection depends mainly on experts' involvement; thus, results are based on personal opinions, knowledge, and judgment. However, to avoid this limitation, a committee of 15 experts was utilized to provide different preferences. Other multicriteria evaluation techniques (ELECTRE, ANP, VIKOR, PROMETHEE, etc.) could be employed to achieve the same goal, and findings could be compared. The analysis's weakness could also be strengthened by including novel criteria. Moreover, we did not use geographic information systems (GIS) to locate candidate sites for OWPS^68–70^, which is an issue to be addressed in future work.

## Supplementary Information

Supplementary Tables.
