# Supplementary material for: Offshore wind power station (OWPS) site selection using a two-stage MCDM-based spherical fuzzy set approach
Source: Sci Rep. 2022 Mar 11;12:4260. doi: 10.1038/s41598-022-08257-2 (PMC8917154; doi:10.1038/s41598-022-08257-2)
Supplement: Supplementary file 1 — Supplementary Tables. [file 41598_2022_8257_MOESM1_ESM.docx]

Article

Offshore Wind Power Station (OWPS) Site Selection Using a Two-Stage MCDM-Based Spherical Fuzzy Set Approach

Chia-Nan Wang ^1^, Ngoc-Ai-Thy Nguyen ^1,^* and Thanh-Tuan Dang ^1,2^

^1^ Department of Industrial Engineering and Management, National Kaohsiung University of Science and Technology, Kaohsiung 80778, Taiwan; [cn.wang@nkust.edu.tw](mailto:cn.wang@nkust.edu.tw) (C.-N.W.); [tuandang.ise@gmail.com](mailto:tuandang.ise@gmail.com) (T.-T.D.)

^2^ Department of Logistics and Supply Chain Management, Hong Bang International University,
Ho Chi Minh 723000, Vietnam

***** Correspondence: [thy.logistics@gmail.com](mailto:thy.logistics@gmail.com) (N.-A.-T.N.)

Appendix A

**Table A1.** The integrated spherical fuzzy comparison matrix of the SF-AHP model.

|  | **C11** | | | **C12** | | | **C21** | | | **C22** | | | **C31** | | |
| --- | --- | --- | --- | --- | --- | --- | --- | --- | --- | --- | --- | --- | --- | --- | --- |
|  | $\boldsymbol{\mu}$ | $\boldsymbol{v}$ | $\boldsymbol{\pi}$ | $\boldsymbol{\mu}$ | $\boldsymbol{v}$ | $\boldsymbol{\pi}$ | $\boldsymbol{\mu}$ | $\boldsymbol{v}$ | $\boldsymbol{\pi}$ | $\boldsymbol{\mu}$ | $\boldsymbol{v}$ | $\boldsymbol{\pi}$ | $\boldsymbol{\mu}$ | $\boldsymbol{v}$ | $\boldsymbol{\pi}$ |
| C11 | 0.500 | 0.400 | 0.400 | 0.557 | 0.415 | 0.321 | 0.470 | 0.504 | 0.331 | 0.546 | 0.446 | 0.304 | 0.461 | 0.534 | 0.293 |
| C12 | 0.391 | 0.584 | 0.310 | 0.500 | 0.400 | 0.400 | 0.493 | 0.482 | 0.336 | 0.424 | 0.576 | 0.275 | 0.519 | 0.476 | 0.288 |
| C21 | 0.466 | 0.497 | 0.336 | 0.458 | 0.511 | 0.430 | 0.500 | 0.400 | 0.400 | 0.596 | 0.391 | 0.297 | 0.435 | 0.556 | 0.300 |
| C22 | 0.402 | 0.589 | 0.289 | 0.503 | 0.490 | 0.216 | 0.331 | 0.660 | 0.262 | 0.500 | 0.400 | 0.400 | 0.390 | 0.604 | 0.280 |
| C31 | 0.480 | 0.510 | 0.298 | 0.403 | 0.585 | 0.182 | 0.399 | 0.590 | 0.282 | 0.557 | 0.429 | 0.302 | 0.500 | 0.400 | 0.400 |
| C32 | 0.373 | 0.613 | 0.283 | 0.442 | 0.523 | 0.234 | 0.381 | 0.603 | 0.296 | 0.445 | 0.536 | 0.314 | 0.523 | 0.455 | 0.313 |
| C33 | 0.541 | 0.447 | 0.305 | 0.547 | 0.432 | 0.488 | 0.458 | 0.511 | 0.338 | 0.594 | 0.394 | 0.289 | 0.594 | 0.392 | 0.287 |
| C41 | 0.551 | 0.430 | 0.308 | 0.578 | 0.405 | 0.471 | 0.537 | 0.439 | 0.313 | 0.582 | 0.419 | 0.276 | 0.512 | 0.492 | 0.276 |
| C42 | 0.498 | 0.493 | 0.294 | 0.536 | 0.441 | 0.297 | 0.406 | 0.580 | 0.293 | 0.531 | 0.452 | 0.313 | 0.562 | 0.413 | 0.306 |
| C51 | 0.384 | 0.607 | 0.282 | 0.512 | 0.470 | 0.179 | 0.357 | 0.635 | 0.269 | 0.485 | 0.507 | 0.294 | 0.462 | 0.512 | 0.318 |
| C52 | 0.300 | 0.690 | 0.250 | 0.503 | 0.476 | 0.258 | 0.498 | 0.479 | 0.321 | 0.421 | 0.560 | 0.310 | 0.470 | 0.506 | 0.332 |
| C53 | 0.381 | 0.589 | 0.316 | 0.450 | 0.543 | 0.142 | 0.331 | 0.660 | 0.262 | 0.458 | 0.511 | 0.338 | 0.361 | 0.613 | 0.302 |
| C61 | 0.542 | 0.436 | 0.311 | 0.508 | 0.473 | 0.325 | 0.458 | 0.511 | 0.338 | 0.571 | 0.416 | 0.299 | 0.541 | 0.427 | 0.322 |
| C62 | 0.429 | 0.554 | 0.303 | 0.517 | 0.461 | 0.407 | 0.536 | 0.441 | 0.315 | 0.634 | 0.365 | 0.261 | 0.594 | 0.394 | 0.289 |
| C63 | 0.484 | 0.495 | 0.318 | 0.450 | 0.543 | 0.155 | 0.395 | 0.596 | 0.285 | 0.488 | 0.493 | 0.316 | 0.458 | 0.534 | 0.291 |
|  | **C32** | | | **C33** | | | **C41** | | | **C42** | | | **C51** | | |
| C11 | 0.565 | 0.421 | 0.305 | 0.398 | 0.599 | 0.280 | 0.368 | 0.628 | 0.272 | 0.441 | 0.556 | 0.286 | 0.567 | 0.424 | 0.299 |
| C12 | 0.496 | 0.476 | 0.332 | 0.386 | 0.607 | 0.279 | 0.373 | 0.621 | 0.273 | 0.404 | 0.586 | 0.293 | 0.419 | 0.573 | 0.289 |
| C21 | 0.569 | 0.412 | 0.311 | 0.493 | 0.482 | 0.336 | 0.407 | 0.582 | 0.293 | 0.529 | 0.459 | 0.304 | 0.593 | 0.397 | 0.291 |
| C22 | 0.500 | 0.486 | 0.314 | 0.340 | 0.659 | 0.252 | 0.319 | 0.685 | 0.228 | 0.386 | 0.612 | 0.278 | 0.453 | 0.544 | 0.285 |
| C31 | 0.418 | 0.571 | 0.293 | 0.346 | 0.650 | 0.253 | 0.414 | 0.592 | 0.257 | 0.378 | 0.612 | 0.279 | 0.472 | 0.511 | 0.314 |
| C32 | 0.500 | 0.400 | 0.400 | 0.493 | 0.482 | 0.336 | 0.431 | 0.562 | 0.289 | 0.471 | 0.508 | 0.328 | 0.629 | 0.367 | 0.273 |
| C33 | 0.458 | 0.511 | 0.338 | 0.500 | 0.400 | 0.400 | 0.493 | 0.482 | 0.336 | 0.450 | 0.531 | 0.321 | 0.585 | 0.406 | 0.295 |
| C41 | 0.498 | 0.484 | 0.308 | 0.458 | 0.511 | 0.338 | 0.500 | 0.400 | 0.400 | 0.493 | 0.482 | 0.336 | 0.619 | 0.356 | 0.298 |
| C42 | 0.475 | 0.494 | 0.336 | 0.493 | 0.475 | 0.332 | 0.458 | 0.511 | 0.338 | 0.500 | 0.400 | 0.400 | 0.659 | 0.342 | 0.254 |
| C51 | 0.331 | 0.665 | 0.249 | 0.331 | 0.662 | 0.255 | 0.338 | 0.647 | 0.276 | 0.288 | 0.710 | 0.216 | 0.500 | 0.400 | 0.400 |
| C52 | 0.432 | 0.549 | 0.310 | 0.421 | 0.560 | 0.310 | 0.384 | 0.598 | 0.296 | 0.338 | 0.649 | 0.269 | 0.536 | 0.450 | 0.307 |
| C53 | 0.338 | 0.649 | 0.269 | 0.325 | 0.675 | 0.241 | 0.425 | 0.550 | 0.314 | 0.373 | 0.619 | 0.275 | 0.429 | 0.544 | 0.324 |
| C61 | 0.489 | 0.491 | 0.314 | 0.466 | 0.505 | 0.329 | 0.475 | 0.494 | 0.336 | 0.446 | 0.509 | 0.352 | 0.512 | 0.464 | 0.321 |
| C62 | 0.536 | 0.446 | 0.301 | 0.541 | 0.447 | 0.305 | 0.498 | 0.472 | 0.331 | 0.446 | 0.532 | 0.307 | 0.507 | 0.483 | 0.304 |
| C63 | 0.347 | 0.633 | 0.283 | 0.381 | 0.605 | 0.289 | 0.399 | 0.576 | 0.316 | 0.354 | 0.636 | 0.275 | 0.391 | 0.584 | 0.310 |
|  | **C52** | | | **C53** | | | **C61** | | | **C62** | | | **C63** | | |
| C11 | 0.641 | 0.337 | 0.287 | 0.566 | 0.397 | 0.328 | 0.396 | 0.595 | 0.286 | 0.509 | 0.479 | 0.303 | 0.460 | 0.528 | 0.311 |
| C12 | 0.435 | 0.556 | 0.300 | 0.479 | 0.522 | 0.281 | 0.431 | 0.559 | 0.296 | 0.423 | 0.567 | 0.300 | 0.479 | 0.522 | 0.281 |
| C21 | 0.447 | 0.541 | 0.307 | 0.596 | 0.391 | 0.297 | 0.493 | 0.482 | 0.336 | 0.404 | 0.586 | 0.293 | 0.550 | 0.443 | 0.296 |
| C22 | 0.529 | 0.454 | 0.318 | 0.493 | 0.482 | 0.336 | 0.327 | 0.674 | 0.245 | 0.305 | 0.696 | 0.220 | 0.448 | 0.543 | 0.303 |
| C31 | 0.486 | 0.497 | 0.329 | 0.576 | 0.389 | 0.324 | 0.388 | 0.599 | 0.292 | 0.340 | 0.659 | 0.252 | 0.479 | 0.518 | 0.288 |
| C32 | 0.515 | 0.469 | 0.318 | 0.608 | 0.373 | 0.297 | 0.451 | 0.538 | 0.303 | 0.390 | 0.604 | 0.276 | 0.595 | 0.377 | 0.307 |
| C33 | 0.529 | 0.454 | 0.318 | 0.617 | 0.385 | 0.274 | 0.476 | 0.504 | 0.324 | 0.398 | 0.599 | 0.280 | 0.559 | 0.426 | 0.309 |
| C41 | 0.570 | 0.410 | 0.309 | 0.513 | 0.467 | 0.317 | 0.471 | 0.508 | 0.328 | 0.438 | 0.546 | 0.313 | 0.552 | 0.421 | 0.325 |
| C42 | 0.608 | 0.373 | 0.297 | 0.572 | 0.421 | 0.297 | 0.495 | 0.468 | 0.349 | 0.480 | 0.505 | 0.303 | 0.603 | 0.383 | 0.296 |
| C51 | 0.409 | 0.586 | 0.287 | 0.517 | 0.459 | 0.329 | 0.435 | 0.553 | 0.307 | 0.433 | 0.564 | 0.290 | 0.557 | 0.415 | 0.321 |
| C52 | 0.500 | 0.400 | 0.400 | 0.444 | 0.531 | 0.327 | 0.441 | 0.563 | 0.264 | 0.477 | 0.515 | 0.300 | 0.571 | 0.421 | 0.291 |
| C53 | 0.493 | 0.466 | 0.340 | 0.500 | 0.400 | 0.400 | 0.375 | 0.621 | 0.278 | 0.378 | 0.612 | 0.279 | 0.568 | 0.406 | 0.314 |
| C61 | 0.472 | 0.527 | 0.273 | 0.545 | 0.436 | 0.313 | 0.500 | 0.400 | 0.400 | 0.493 | 0.482 | 0.336 | 0.552 | 0.421 | 0.325 |
| C62 | 0.454 | 0.530 | 0.308 | 0.562 | 0.413 | 0.306 | 0.458 | 0.511 | 0.338 | 0.500 | 0.400 | 0.400 | 0.637 | 0.367 | 0.263 |
| C63 | 0.377 | 0.613 | 0.278 | 0.373 | 0.605 | 0.296 | 0.399 | 0.576 | 0.316 | 0.331 | 0.669 | 0.242 | 0.500 | 0.400 | 0.400 |

**Table A2.** The weighted normalized matrix for WSM of the WASPAS model.

| **DMU** | **Location** | **C11** | **C12** | **C21** | **C22** | **C31** | **C32** | **C33** | **C41** |
| --- | --- | --- | --- | --- | --- | --- | --- | --- | --- |
| OWPS-01 | Ba Ria - Vung Tau | 0.048 | 0.016 | 0.031 | 0.018 | 0.019 | 0.026 | 0.032 | 0.034 |
| OWPS-02 | Ben Tre | 0.048 | 0.015 | 0.040 | 0.025 | 0.031 | 0.040 | 0.044 | 0.034 |
| OWPS-03 | Binh Dinh | 0.048 | 0.014 | 0.051 | 0.036 | 0.043 | 0.052 | 0.053 | 0.051 |
| OWPS-04 | Binh Thuan | 0.063 | 0.045 | 0.048 | 0.059 | 0.062 | 0.069 | 0.073 | 0.074 |
| OWPS-05 | Ca Mau | 0.045 | 0.013 | 0.011 | 0.011 | 0.012 | 0.011 | 0.009 | 0.017 |
| OWPS-06 | Ninh Thuan | 0.071 | 0.062 | 0.071 | 0.045 | 0.048 | 0.057 | 0.050 | 0.054 |
| OWPS-07 | Soc Trang | 0.048 | 0.014 | 0.051 | 0.032 | 0.038 | 0.049 | 0.053 | 0.051 |
| **DMU** | **Location** | **C42** | **C51** | **C52** | **C53** | **C61** | **C62** | **C63** |  |
| OWPS-01 | Ba Ria - Vung Tau | 0.030 | 0.040 | 0.025 | 0.027 | 0.019 | 0.041 | 0.019 |  |
| OWPS-02 | Ben Tre | 0.045 | 0.019 | 0.036 | 0.025 | 0.022 | 0.031 | 0.040 |  |
| OWPS-03 | Binh Dinh | 0.060 | 0.014 | 0.047 | 0.033 | 0.041 | 0.022 | 0.050 |  |
| OWPS-04 | Binh Thuan | 0.075 | 0.011 | 0.064 | 0.058 | 0.071 | 0.014 | 0.057 |  |
| OWPS-05 | Ca Mau | 0.015 | 0.060 | 0.011 | 0.009 | 0.019 | 0.075 | 0.010 |  |
| OWPS-06 | Ninh Thuan | 0.060 | 0.012 | 0.058 | 0.051 | 0.054 | 0.018 | 0.055 |  |
| OWPS-07 | Soc Trang | 0.045 | 0.014 | 0.044 | 0.033 | 0.041 | 0.025 | 0.038 |  |

**Table A3.** Exponentially weighted normalized matrix for WPM of the WASPAS model.

| **DMU** | **Location** | **C11** | **C12** | **C21** | **C22** | **C31** | **C32** | **C33** | **C41** |
| --- | --- | --- | --- | --- | --- | --- | --- | --- | --- |
| OWPS-01 | Ba Ria - Vung Tau | 0.973 | 0.918 | 0.944 | 0.933 | 0.929 | 0.935 | 0.942 | 0.945 |
| OWPS-02 | Ben Tre | 0.972 | 0.916 | 0.960 | 0.951 | 0.958 | 0.964 | 0.963 | 0.945 |
| OWPS-03 | Binh Dinh | 0.972 | 0.913 | 0.977 | 0.972 | 0.977 | 0.980 | 0.976 | 0.973 |
| OWPS-04 | Binh Thuan | 0.991 | 0.980 | 0.973 | 1.000 | 1.000 | 1.000 | 1.000 | 1.000 |
| OWPS-05 | Ca Mau | 0.969 | 0.908 | 0.878 | 0.908 | 0.902 | 0.884 | 0.856 | 0.898 |
| OWPS-06 | Ninh Thuan | 1.000 | 1.000 | 1.000 | 0.985 | 0.984 | 0.988 | 0.972 | 0.977 |
| OWPS-07 | Soc Trang | 0.972 | 0.913 | 0.977 | 0.964 | 0.970 | 0.977 | 0.976 | 0.973 |
| **DMU** | **Location** | **C42** | **C51** | **C52** | **C53** | **C61** | **C62** | **C63** |  |
| OWPS-01 | Ba Ria - Vung Tau | 0.934 | 0.976 | 0.942 | 0.956 | 0.911 | 0.957 | 0.939 |  |
| OWPS-02 | Ben Tre | 0.962 | 0.931 | 0.964 | 0.951 | 0.920 | 0.937 | 0.981 |  |
| OWPS-03 | Binh Dinh | 0.983 | 0.916 | 0.981 | 0.969 | 0.962 | 0.913 | 0.992 |  |
| OWPS-04 | Binh Thuan | 1.000 | 0.902 | 1.000 | 1.000 | 1.000 | 0.884 | 1.000 |  |
| OWPS-05 | Ca Mau | 0.887 | 1.000 | 0.895 | 0.897 | 0.911 | 1.000 | 0.903 |  |
| OWPS-06 | Ninh Thuan | 0.983 | 0.907 | 0.994 | 0.993 | 0.982 | 0.898 | 0.998 |  |
| OWPS-07 | Soc Trang | 0.962 | 0.916 | 0.977 | 0.969 | 0.962 | 0.921 | 0.977 |  |
